# *LRRK2* G2019S variant is associated with transcriptional changes in Parkinson’s disease human myeloid cells under proinflammatory environment

**DOI:** 10.1186/s12974-026-03967-0

**Published:** 2026-08-08

**Authors:** Elisa Navarro, Anastasia G. Efthymiou, Daniele Mattei, Madison Parks, Giulietta Maria Riboldi, Ricardo A. Vialle, Evan Udine, Benjamin Z. Muller, Jack Humphrey, Amanda Allan, Charalambos Argyrou, Katia de Paiva Lopes, Alexandra Münch, Deborah Raymond, Rivka Sachdev, Vicki L. Shanker, Joan Miravite, Viktoriya Katsnelson, Katherine Leaver, Steven Frucht, Susan B. Bressman, Edoardo Marcora, Rachel Saunders-Pullman, Alison M. Goate, Towfique Raj

**Affiliations:** 1 Nash Family Department of Neuroscience & Friedman Brain Institute, Icahn School of Medicine at Mount Sinai, New York, NY United States of America; 2 Ronald M. Loeb Center for Alzheimer’s Disease, Icahn School of Medicine at Mount Sinai, New York, NY United States of America; 3 Department of Genetics and Genomic Sciences & Icahn Genomics Institute, Icahn School of Medicine at Mount Sinai, New York, NY United States of America; 4 Estelle and Daniel Maggin Department of Neurology, Icahn School of Medicine at Mount Sinai, New York, NY United States of America; 5 Departamento de Bioquimica y Biologia Molecular, Facultad de Medicina, Instituto Universitario de Investigacion en Neuroquimica, Universidad Complutense, Madrid, Spain; 6 Centro de Investigacion Biomedica en Red de Enfermedades Neurodegenerativas (CIBERNED), Instituto de Salud Carlos III, Madrid, Spain; 7 Instituto Ramon y Cajal de Investigacion Sanitaria (IRYCIS), Madrid, Spain; 8 New York University Grossman School of Medicine, New York, NY United States of America; 9 Rush Alzheimer’s Disease Center, Rush University Medical Center, Chicago, IL 60612 United States of America; 10 Department of Neurological Sciences, Rush University Medical Center, Chicago, IL 60612 United States of America; 11 Bonnie and Tom Strauss Center for Movement Disorders, Clinical Neurosciences Center, Department of Neurology, Icahn School of Medicine at Mount Sinai, New York, NY United States of America

## Abstract

The G2019S mutation in the leucine-rich repeat kinase 2 (LRRK2) gene is a major risk factor for the development of Parkinson’s disease (PD). LRRK2, although expressed in different cell types, is highly abundant in cells of the innate immune system. Given the importance of central and peripheral immune cells in the development of PD, we sought to investigate the consequences of the G2019S mutation on microglial and monocyte transcriptome and function. We have generated large-scale transcriptomic profiles of isogenic human induced microglial cells (iMGLs) and patient derived monocytes carrying the G2019S mutation in untreated culture conditions and following exposure to the proinflammatory factors IFNγ and LPS. We demonstrate that the G2019S mutation exerts a profound impact on the transcriptomic profile of these myeloid cells, and describe corresponding functional differences in iMGLs. The G2019S mutation led to an upregulation in lipid metabolism and phagolysosomal pathway genes in untreated and LPS/IFNγ stimulated iMGLs, which was accompanied by an increased phagocytic capacity of myelin debris. We also identified dysregulation of cell cycle genes, with a downregulation of the E2F4 regulon. Transcriptomic characterization of human-derived monocytes carrying the G2019S mutation confirmed alteration in lipid metabolism associated genes. Altogether, these findings reveal the influence of G2019S on the dysregulation of the myeloid cell transcriptome under proinflammatory conditions.

**Supplementary Information:**

The online version contains supplementary material available at https://doi.org/10.1186/s12974-026-03967-0.

## Introduction

Parkinson’s disease (PD) is a progressive neurodegenerative disorder with no cure and no approved disease-modifying treatments. PD is highly heritable, and early studies of PD focused on causal genes implicated in familial PD including *SNCA*,* GBA*,* and LRRK2* [1–4]. More recently, genome-wide association studies (GWAS) have identified 90 independent genetic loci across 78 genomic regions that contribute to sporadic forms of the disease, and approximately 15% of people with PD, but without risk variants in PD associated genes, have family history of PD in first-degree relatives. These findings provide compelling evidence for the existence of a heritable component in sporadic PD [5]. We have previously demonstrated that PD risk alleles are expression quantitative trait loci (eQTLs) in peripheral monocytes and that monocytes and microglia derived from PD patients show profound transcriptional dysregulation [6–8]. Data from other groups have also shown an enrichment of PD heritability in immune cell types, suggesting that PD risk genes play a major role within these cell types [6–12].

GWAS have identified PD-associated loci harboring genes known to be involved in familial PD, indicating a potential shared molecular basis between sporadic and familial forms of the disease [13]. *LRRK2* is one such gene, with common and rare variants that confer a modest to high risk for developing PD [8, 13–15]. These *LRRK2* variants have been shown to be associated with a host of alterations in many cellular processes including disordered vesicular trafficking, cytoskeletal dynamics, mitochondrial function, autophagy and lysosomal function, as well as immune system dysregulation [16]. While numerous single nucleotide polymorphisms (SNPs) have been discovered within the *LRRK2* locus, the most prevalent variant associated with PD involves a Glycine to Serine substitution at amino acid position 2019 (G2019S), which is within the kinase domain and is thought to increase kinase activity [17]. *LRRK2* G2019S-linked PD (LRRK2-PD) exhibits clinical features that are indistinguishable from idiopathic PD, has increasing penetrance with age, and accounts for 1–5% of sporadic cases of PD and 1–18% of familial cases depending on ancestral population [18]. Due to the genetic overlap between LRRK2-PD and other familial PD, as well as the similar clinical presentation of patients between LRRK2-PD and idiopathic PD, studying the role of LRRK2 in PD is critical both for understanding disease etiology as well as developing targets for therapeutic intervention.

LRRK2 is expressed in different cell types within the brain, and much research has focused on the role of the G2019S mutation on neuronal and astrocytic function [16]. However, the highest LRRK2 expression is seen in immune cells, particularly peripheral blood mononuclear cells (PBMCs), and this gene seems to play a pivotal role in immune regulation. LRRK2 has been shown to be associated with the activation and maturation of human monocytes [19] and in modulating neuroinflammation by cytokine signaling [20, 21]. Variants in *LRRK2* have been implicated in different immune-related disorders including Crohn’s disease and leprosy [22], or cancer [23], while elimination of the *LRRK2* gene seems to suppress immune system activation [21]. *LRRK2* risk variants have been reported to be associated with higher levels of LRRK2 expression in monocytes [8] and monocyte-derived microglia [24]. Moreover, *LRRK2* expression is highly dependent on inflammatory state [19, 24–27], and has been shown to respond to different proinflammatory stimuli such as interferon-γ (IFNγ) [25, 27], lipopolysaccharides (LPS) [28], IFNβ, tumor necrosis factor α (TNFα), and interleukin-6 (IL-6) [19]. Finally, it has also been reported that non-coding common genetic variants influence *LRRK2* expression specifically in microglia, pointing to the cellular specificity of genetic variation and highlighting the role of microglia in the context of *LRRK2 *[29].

Collectively, these data suggest that there may be an association between the innate immune system and PD, especially in PD patients carrying mutations in *LRRK2*. Due to the reduced penetrance of disease onset in LRRK2 carriers, we hypothesize that *LRRK2* variants affect innate immune cells, such as monocytes, macrophages, and microglia, in proinflammatory conditions to affect disease onset and progression. Thus, our objective is to characterize and understand the transcriptional and functional effect of the G2019S variant in myeloid cells. Here, we have used human induced microglial cells as well as patient-derived monocytes and characterized the G2019S effect in untreated cells and following stimulation with the proinflammatory regulators IFNγ and LPS. Our findings demonstrate that the G2019S mutation has a strong influence in myeloid cells transcriptome and functionality, affecting phagocytosis, lipid metabolism, and proliferation genes.

## Results

### Generation of isogenic *LRRK2* G2019S iPSC-derived microglia

**Fig. 1:**
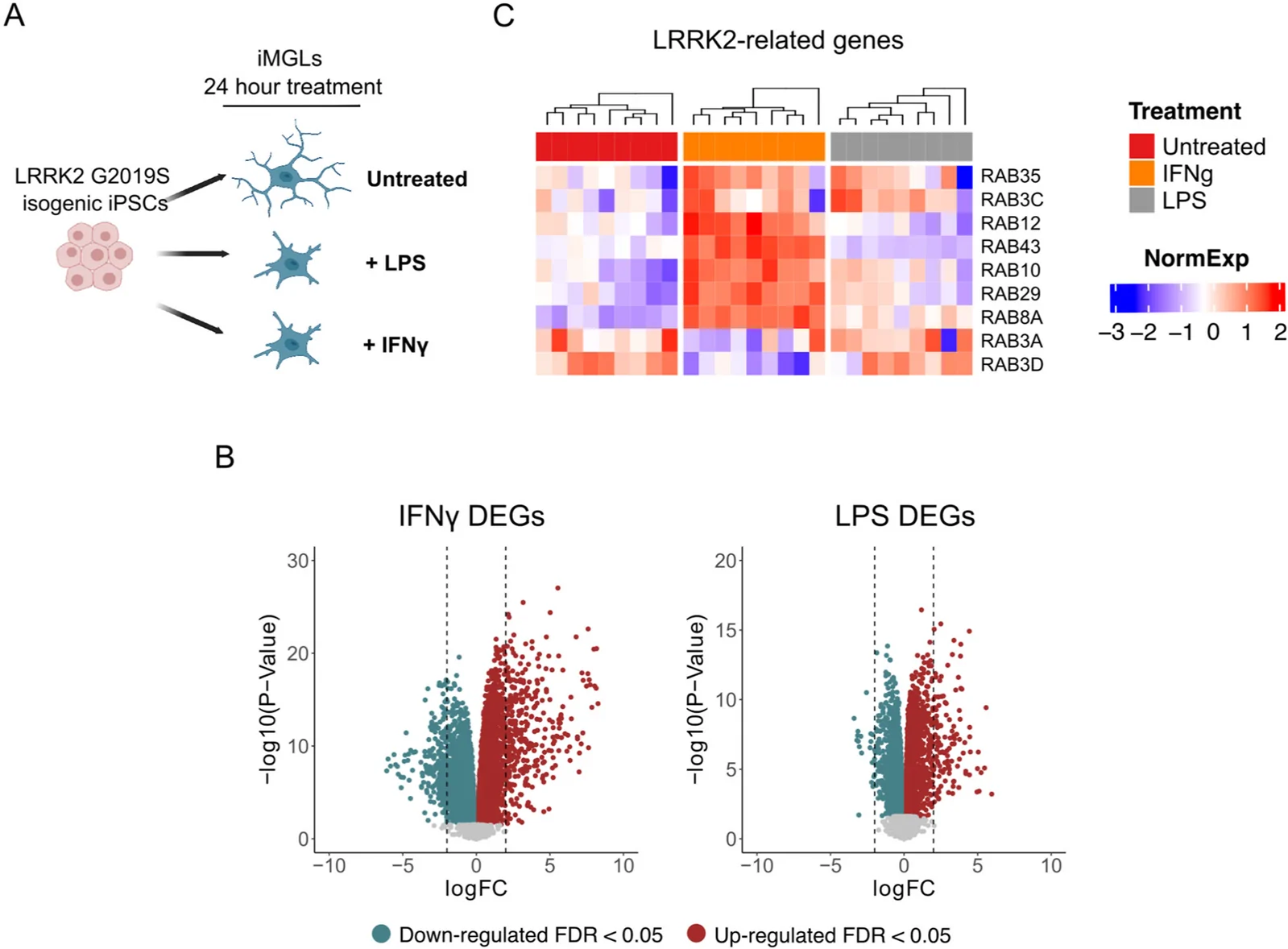
Human induced microglial cells respond to IFNγ and LPS. **A** Overview of the study design. CRISPR-edited isogenic induced pluripotent stem cells for the G2019S mutation in the *LRRK2* gene were differentiated to microglial cells (iMGLs) and stimulated with IFNγ or LPS for 24 h. **B** Volcano plots showing differential expression after stimulation with IFNγ (left) or LPS (right). Plots show the FC of genes (x axis, log_2_ scale) and their *P* value significance (y axis, –log_10_ scale). Differentially expressed genes (DEGs) at FDR < 0.05 are highlighted in red (upregulated genes) and blue (downregulated genes). Dashed lines show logFC cut-off of 2. A moderated *t*-statistic (two-sided) was used as the statistical test. **C** Heatmap showing the normalized expression of a subset of *LRRK2*-interactors (y axis) across samples (x axis), clustered by treatment. Blue represents normalized expression < 0 and red represents normalized expression > 0

We investigated the impact of proinflammatory stimuli in microglia, brain-resident macrophages that express LRRK2 and are implicated in PD. To reduce variability and enhance statistical power with small sample size, we utilized an isogenic induced pluripotent stem cell (iPSC) line differentiated to induced microglial cells (iMGLs). We obtained three isogenic CRISPR-edited iPSC clonal lines from a single female donor, one carrying wild-type (WT), one carrying heterozygous (het), and one carrying homozygous (hom) *LRRK2* G2019S through the Cookson laboratory [30]. We differentiated these iPSCs to iMGLs using a previously described protocol [31] and treated them with 20 ng/mL IFNγ or 10 ng/mL LPS for 24 h, following a similar pipeline as other authors using human myeloid cells [32] (Fig. 1A). Stimulation of WT iMGLs with IFNγ or LPS showed increased expression of *IFIT1*, *IL1B*, and *TNFa* compared to untreated WT iMGLs (Fig. S1B). IFNγ treatment also induced an increase in expression of LRRK2, which has also been previously described in the literature [19]. For downstream analyses, we collected data from three independent differentiations of each clonal line followed by 24 h of treatment (untreated, LPS and IFNγ), yielding a total of 27 samples (Supplementary Table 1).

### iMGLs respond to IFNγ and LPS with profound transcriptomic changes and cytokine release

We examined the effect of proinflammatory stimuli (IFNγ and LPS) on the iMGL transcriptome by comparing treated and untreated samples, irrespective of genotype. Both stimuli induced substantial changes in gene expression, with IFNγ eliciting more DEGs (598 at FDR < 0.05 and logFC > 2) than LPS (141 at FDR < 0.05 and logFC > 2) (Fig. 1B, Supplementary Tables 2 and 3). Despite differences in DEG counts, there was significant overlap in the responses to the two stimuli (*p*-value = 2.2e-138, Odds ratio = 2.6, overlap tested using Fisher’s exact test) [33] (Fig. 1B). We also assessed whether changes in gene expression corresponded to altered cytokine profiles in iMGLs. Stimulation with IFNγ and LPS significantly increased the secretion of multiple proinflammatory cytokines, including IL-12p40, IL-15, IL-1RA, and TNFa (Supplementary Table 4, two-way ANOVA with Dunnett’s post hoc test, *P* < 0.05), demonstrating that iMGLs respond transcriptionally and functionally to proinflammatory stimuli.

Gene set enrichment analysis (GSEA) using Gene Ontology: Biological Processes (GO: BP) revealed upregulation of immune response pathways and downregulation of protein localization, transcription, and translation pathways (Fig. S1C). The predominant auto-annotated clusters involved an upregulation of immune response pathways, as expected. Additionally, enrichment analysis of 407 potential LRRK2 interactors showed significant enrichment for LRRK2-associated genes in response to both IFNγ and LPS (Fisher’s exact test, IFNγ *P* = 0.0146; LPS *P* = 0.00012, Fig. 1C). These findings indicate that both stimuli induce transcriptional changes relevant to LRRK2, supporting their use in downstream experiments.

### Transcriptional dysregulation in LRRK2-G2019S iMGLs under proinflammatory stimuli

**Fig. 2:**
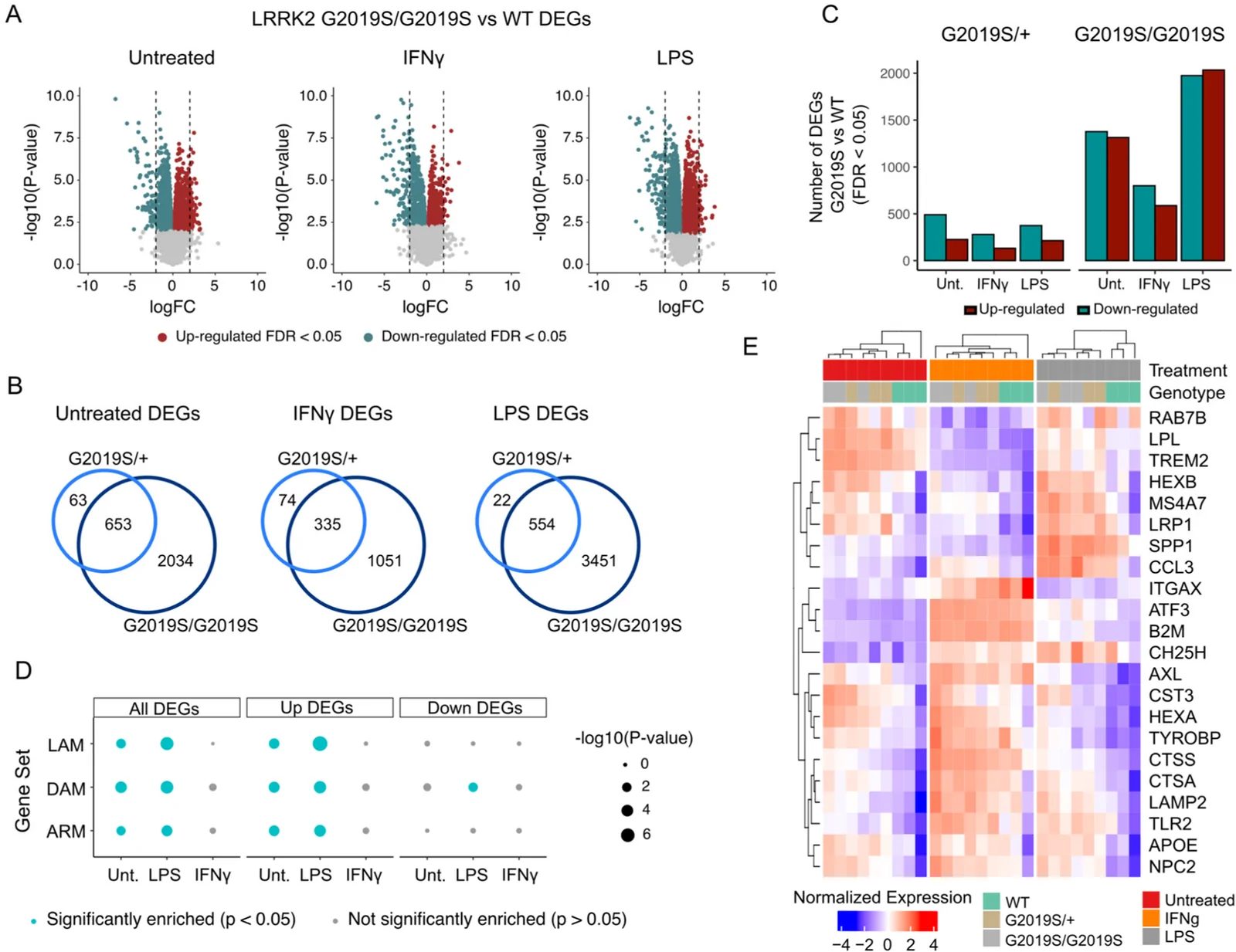
The G2019S mutation drives transcriptomic dysregulation in iMGLs upon IFNγ and LPS stimulation. **A** Volcano plots showing differential expression between LRRK2-G2019S/G2019S (homs) and LRRK2-WT in untreated condition and after stimulation with IFNγ or LPS. Plots show the FC of genes (x axis, log_2_ scale) and their *P* value significance (y axis, –log_10_ scale). DEGs at FDR < 0.05 are highlighted in red (upregulated genes) and blue (downregulated genes). Dashed lines show logFC cut-off of 2. A moderated *t*-statistic (two-sided) was used as the statistical test. **B** Venn diagrams showing the number of shared DEGs between heterozygous (G2019S/+ iMGLs and homozygous iMGLs (G2019S/G2019S) vs. LRRK2-WT. **C** Barplot showing the number of DEGs between LRRK2-G2019S (hets and homs) and LRRK2-WT iMGLs under different stimulations. Red bars represent the number of upregulated DEGs (FC > 0; FDR < 0.05) and blue bars represent the number of downregulated DEGs (FC < 0; FDR < 0.05). **D** Enrichment for microglial signatures in G2019S/G2019S iMGLs (homs) in untreated condition and upon stimulation. Blue dots represent microglial phenotypes for which there is a significant enrichment (*P* < 0.05; Fisher’s-exact test) in G2019S/G2019S (homs) transcriptome. DAM = damage-associated microglia; LAM = lipid-accumulating macrophages, ARM = activated response microglia. **E** Heatmap showing the normalized expression of a selection of DEGs in LRRK2-WT, LRRK2-G2019S/+ and LRRK2-G2019S/G2019S. The heatmap shows a selection of DAM/LAM/ARM genes (y axis) across samples (x axis), clustered by treatment. Blue represents normalized expression < 0 and red represents normalized expression > 0

We performed RNA-sequencing on WT and LRRK2-G2019S iMGLs (heterozygous -hets- and homozygous -homs-) with and without IFNγ or LPS stimulation. Consistent with the impact of G2019S on human health outcomes, we obtained a strong transcriptomic dysregulation in LRRK2-G2019S (both hets and homs) compared to WT. We identified a high number of up and down-regulated DEGs (logFC > 2 and FDR < 0.05) in untreated (16 in hets and 143 in homs) and stimulated conditions (LPS: 59 in hets, 132 in homs LRRK2-G2019S; IFNγ: 50 in hets and 107 in homs) (Fig. 2A and C, Supplementary Tables 5–10). Most of DEGs at FDR < 0.05 were shared between hets and homs (91% in untreated, 82% under IFNγ and 94% in LPS) (Fig. 2B), indicating a dosage-dependent effect of the G2019S mutation on gene expression changes (Fig. 2C). Thus, subsequent analyses will be performed considering LRRK2-G2019S hom lines. To assess context-specific effects of G2019S mutation on myeloid cells, we studied the interaction between genotype and treatment. We obtained that 5 genes showed a significant interaction between IFNγ and G2019S mutation (*ITPRIP*, *AKAP2*, *CYSLTR2*, *CXorf21* & *SPOPL*) and 2 genes in LPS condition (*SEPT4* & A*C068946.2*) (Supplementary Tables 11 & 12).

We investigated whether LRRK2-G2019S iMGLs exhibit transcriptional profiles resembling microglial/macrophages subpopulations including disease/damage-associated microglia (DAM) [34–36], activated response microglia (ARM) [37], or lipid-accumulating macrophages (LAM) [38]. Using gene lists for these phenotypes (Supplementary Table 13), we found significantly enrichment for a list of 110 DAM genes in LRRK2-G2019S untreated and LPS-stimulated iMGLs (Fisher’s exact test, Untreated: *P* = 1.16e-4; LPS: *P* = 8.03e-6) (Fig. 2D). Similarly, upregulated DEGs driven by LRRK2-G2019S were enriched for ARM genes [37] (131 genes), in untreated and LPS conditions (Fisher’s exact test, Untreated: *P* = 0.01; LPS: *P* = 4.5e-4) (Fig. 2D). Whether this ARM response is specific to the amyloid plaque deposition model or general protein aggregation and/or other disease aspects remains to be determined. Additionally, LRRK2-G2019S transcriptomic dysregulation was enriched for LAM signature (Fisher’s exact test, Untreated: *P* = 4.6e-3; LPS: *P* = 3.03e-6), reflecting altered lipid homeostasis. Overall, enrichment for these microglial phenotypes was more pronounced in up-regulated DEGs, reflecting that LRRK2-G2019S mutation amplifies these disease-associated signatures. Examples of the effect of LRRK2-G2019S on the expression of specific genes of these signatures is reflected in Fig. 2E.

### Stimulated LRRK2-G2019S iMGLs display an upregulation of phagosome and lipid metabolism genes

**Fig. 3:**
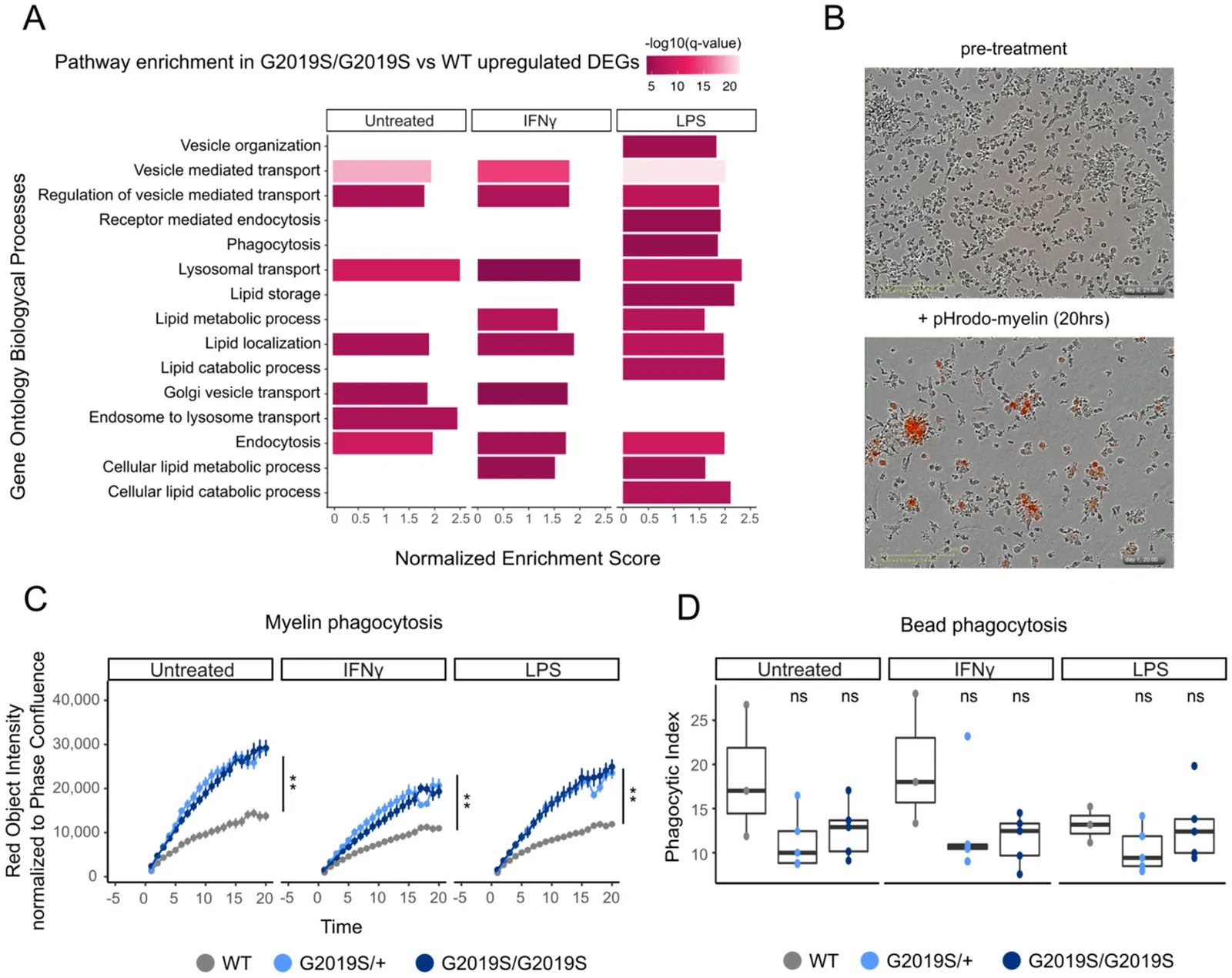
LRRK2-G2019S iMGLs show alterations in lipid metabolism and phago-lysosomal pathways. **A** Pathway enrichment analysis for the upregulated DEGs using Biological Process from GSEA comparing G2019S/G2019S (homs) vs. LRRK2-WT. Pathways are represented in the y axis and normalized score in the x axis. Significance (-log_10_ *P* value scale of the *q*-value) is represented by color. Only significant pathways (*q*-value < 0.05) of pathways related to lipid metabolism and phagocytosis are represented. **B** Representative bright focus microphotographs of iMGLs subjected to ph-rodo labeled human myelin. Top picture shows iMGLs before treatment and bottom picture shows iMGLs after 20 h treatment with pH-rodo labeled human myelin. **C** Myelin phagocytosis of iMGLs under the different treatments measured by IncuCyte microscope. The y axis shows the phagocytic capacity measured as red object intensity normalized by cell confluence and the x axis the different time points. Genotypes begin to diverge in their myelin phagocytosis after 3 h (*n* = 3 independent differentiations, Linear mixed effects model ** *P* value < 0.001) and show significant differences across genotypes by the end of 20 h (*n* = 3 independent differentiations, ANOVA with Dunnett’s post-hoc test ** *P* value < 0.001). **D** Carboxylated-beads phagocytosis of iMGLs under the different treatments measured by FACS. Y axis represents the phagocytic index and x axis the different treatments (*n* = 3 independent differentiations, ANOVA with Dunnett’s post-hoc test)

We performed Gene Set Enrichment Analysis (GSEA) using Gene Ontology (GO) terms on DEGs between LRRK2-G2019S and WT iMGLs under untreated, IFNγ, and LPS conditions to identify affected biological pathways. Upregulated DEGs were significantly enriched in phagocytosis and lipid metabolism pathways (Fig. 3A, Supplementary Table 14). Dysregulated phagocytic genes included *COLEC12* which was strongly upregulated in LRRK2-G2019S cells in the 3 experimental conditions (logFC = 2.26, FDR = 0.0036 in untreated; logFC = 1.57, FDR = 0.0085 in LPS; logFC = 1.95, FDR = 0.014 in INFγ). Other examples are *TREM2* (logFC = 0.99, FDR = 0.01 in LPS) or *AXL* (logFC = 0.45, FDR = 0.005 in untreated; logFC = 0.47, FDR = 0.0015 in LPS). Lipid metabolism dysregulated genes included *LRP1* (logFC = 0.79, FDR = 0.018 in untreated; logFC = 0.66, FDR = 0.02 in LPS, logFC = 0.94, FDR = 0.016 in IFNγ) or *LPL* (logFC = 1.15, FDR = 0.0019 in untreated cells; logFC = 0.98, FDR = 0.0015 in LPS, logFC = 1.14, FDR = 0.0029 in IFNγ). Other examples of phagolysosomal genes significantly up-regulated in LRRK2-G2019S are *CD68*,* FCGR1A*,* FTL*,* GAA*,* GRN* or *TMEM106A*, genes which have also been previously associated with variants in *LRRK2* in a pQTL study of cerebrospinal fluid (CSF) [39]. Together, these data suggest that G2019S mutation drives an increased expression in phagocytosis and lipid metabolism genes.

To assess the functional implications of the transcriptional changes, we performed a phagocytosis assay using pHrodo-conjugated human myelin debris, a lipid-rich substrate. iMGLs were treated with 25 µg of myelin debris for 20 h and imaged hourly to monitor the pHrodo fluorescent signal. LRRK2-G2019S cells (hets and homs) exhibited higher myelin phagocytosis than LRRK2-WT under both untreated and proinflammatory conditions (Fig. 3B, C), as shown by increased red pHrodo signal intensity and object area. To determine if this difference was due to substrate uptake or clearance failure, iMGLs were treated with carboxylated beads and assessed via flow cytometry after 24 h. No differences in bead uptake were observed across genotypes or treatments (Fig. 3D), suggesting that the myelin phagocytosis disparity is not due to uptake variations. Further investigation is required to elucidate the G2019S mutation’s effect on lipid-rich debris processing. These findings, together with transcriptomic data, indicate that differences in myelin clearance are regulated by downstream endolysosomal processes rather than substrate uptake.

### Stimulated LRRK2-G2019S iMGLs display a downregulation of mitosis and cell-cycle genes and decreased proliferation

**Fig. 4:**
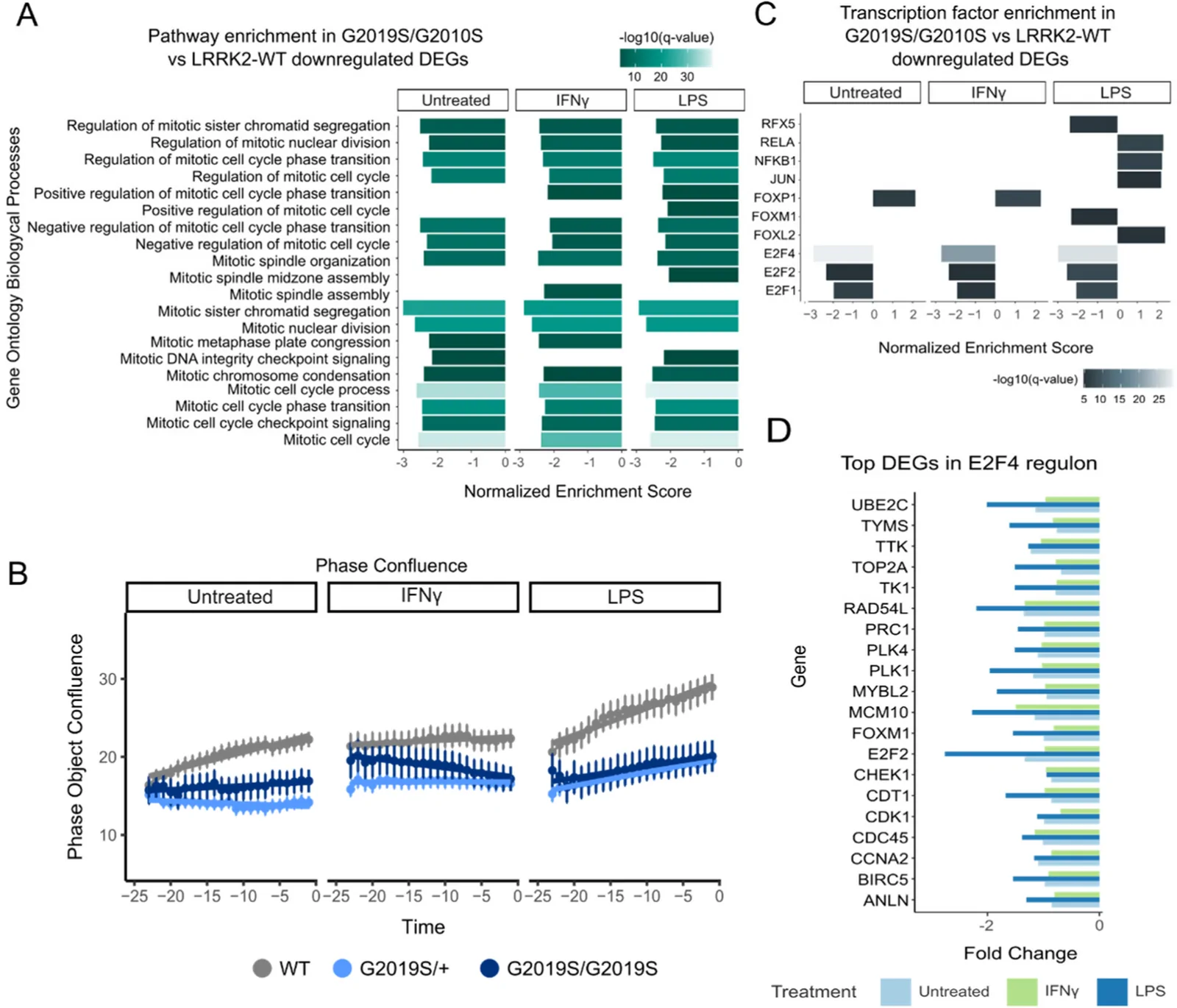
LRRK2-G2019S iMGLs show downregulation in E2F regulon genes. **A** Pathway enrichment analysis for the downregulated DEGs using Biological Process from GSEA comparing G2019S/G2019S (homs) vs. LRRK2-WT. Pathways are represented on the y axis and normalized score on the x axis. Significance (-log_10_ *P* value scale of the q-value) is represented by color. Only significant pathways (*q*-value < 0.05) related to cell mitosis and proliferation are represented. **B** Phase object confluence measurements of iMGLs using IncuCyte live-imaging. The y axis shows the phase object confluence and the x axis represents time, with samples separated by treatment. **C** Transcription factor enrichment using Dorothea comparing G2019S/G2019S (homs) vs. LRRK2-WT. TFs are represented on the y axis and normalized enrichment score on the x axis. Significance (-log10 *P* value scale of the q-value) is represented by color. Only significant regulons (q-value < 0.01) are represented. **D** Boxplot showing fold change of the top 20 DEGs within the *E2F4* regulon under different treatments comparing G2019S/G2019S (homs) vs. LRRK2-WT. Y axis shows gene names and x axis the fold change

We then explored mechanisms associated with downregulated DEGs in LRRK2-G2019S compared to LRRK2-WT iMGLs, and we found a significant enrichment for cell cycle and mitosis pathways under proinflammatory stimuli (Fig. 4A, Supplementary Table 14). To determine whether LRRK2-G2019S iMGLs exhibited deficits in proliferation compared to WT iMGLs, we performed live imaging analysis using IncuCyte and measured phase object confluence of iMGLs over a 24 h period (Fig. 4B). We observed that LRRK2-G2019S iMGLs showed a decreased rate of object confluence over that period, irrespective of treatment, suggesting decreased proliferation. While these data serve as an indirect measure of proliferation, it does support the notion that G2019S iMGLs display diminished proliferation compared to their wild-type counterparts.

We used the regulatory target gene sets list from DoRothEA to identify whether there was an enrichment in particular transcription factor motifs in the LRRK2-G2019S iMGL DEGs that may be mediating the cell cycle and mitosis effects. Interestingly, we observed that LRRK2-G2019S cells displayed a strong downregulation in E2F transcription factor targets in both LPS and IFNγ proinflammatory treatment conditions (Fig. 4C, Supplementary Table 15). The most significant enrichment was observed for E2F4 regulon in which we observed significant downregulation of its target genes without differential expression of E2F4 itself (Fig. 4D).

### G2019S mutation is associated with transcriptomic dysregulation in human derived monocytes

Thus far, our data demonstrate a significant impact of *LRRK2* G2019S genotype and proinflammatory stimuli on the iMGL transcriptome. To extend these findings, we performed a similar analysis on peripheral monocytes isolated from 35 participants divided in 4 groups based on PD diagnosis and *LRRK2* mutation: PD cases without the G2019S mutation (PD-WT), PD cases with the G2019S mutation (PD-G2019S), healthy subjects without the *LRRK2* G2019S mutation (CTRL-WT), and non-PD manifesting carriers of the G2019S variant (CTRL-G2019S). Participants were age-matched and sex balanced (Fig. S3A & B and Supplementary Table 16), and to avoid any effect of age or sex both covariates were regressed out for downstream analyses. Monocytes were treated with LPS and IFNγ for 24 h, followed by RNA-seq.

After extensive quality control (Fig. S4), we performed DE analysis irrespective of disease or LRRK2 mutation genotype and observed that both stimuli, IFNγ and LPS, triggered a profound transcriptional response in human monocytes (Fig. S5A, B, Supplementary Tables 17 & 18). LRRK2 expression increased with IFNγ, but remained unchanged with LPS (Fig. S3C, D), consistent with our iMGL data and previous reports [27, 40]. There were no differences either in LRRK2 expression in carriers vs. non carriers, regardless of PD status (Fig. S3C). Monocytes stimulated with IFNγ and LPS were also enriched for PD and LRRK2-interactor genes (Supplementary Table 13) (Fig. S5C, D). Overall, these results underscore that IFNγ and LPS can produce a proinflammatory response on the human monocyte transcriptome which is relevant in the context of PD.

**Fig. 5:**
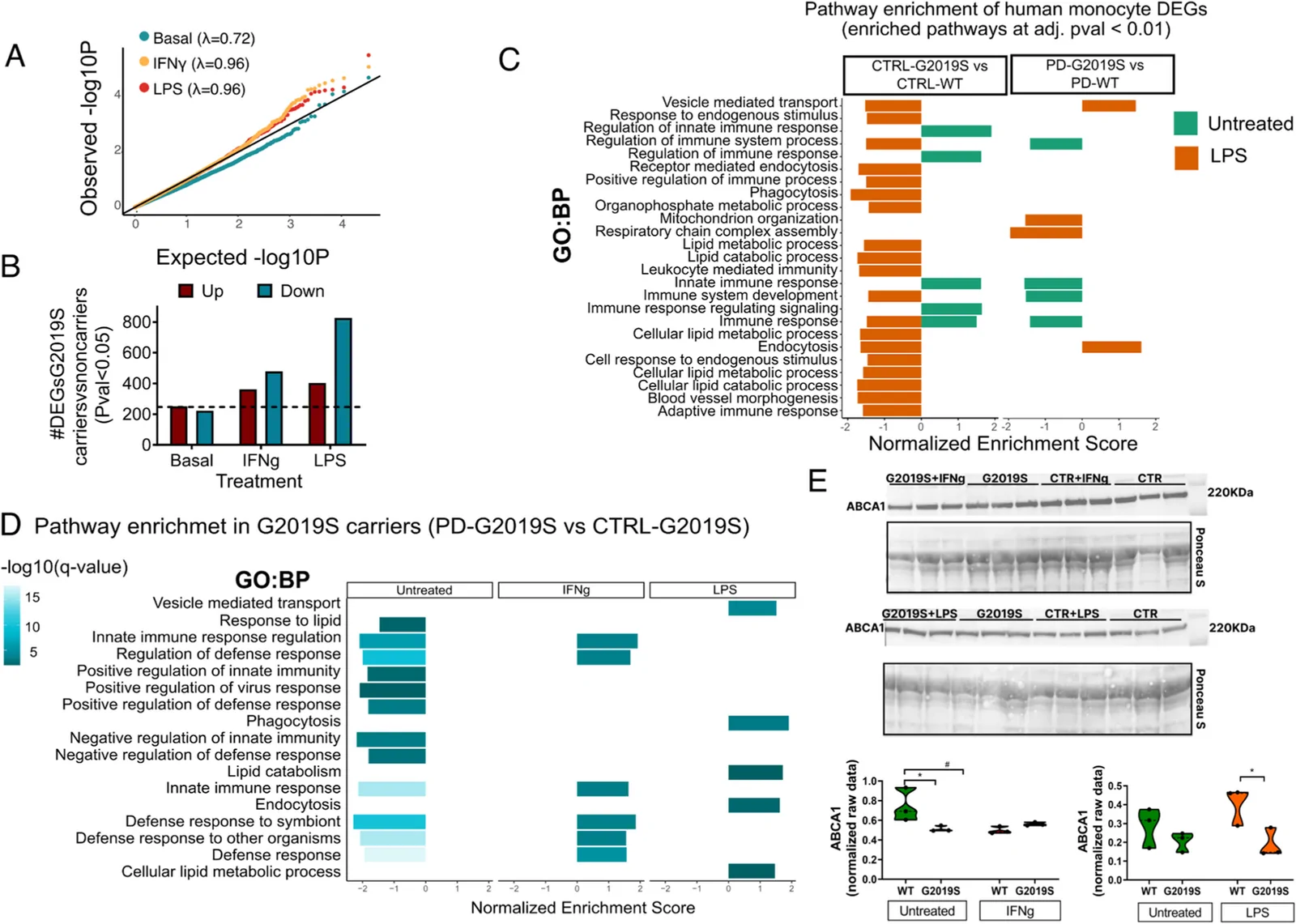
Patient derived monocytes carrying the LRRK2-G2019S mutation show transcriptomic dysregulation. **A** Quantile quantile (qq)-plot shows the observed distribution of *P* values which deviates from the expected uniform distribution across the three different treatments. The genomic inflation factor (λ or X^2^ median) was calculated as the measure of deviations of the observed distribution from the expected null distribution. **B** Barplot showing the number of DEGs at nominal *p*-value between LRRK2-G2019S carriers and LRRK2-WT monocytes under different stimulations. Red bars represent the number of upregulated DEGs (FC > 0; *p*-value < 0.05) and blue bars represent the number of downregulated DEGs (FC < 0; *p*-value < 0.05). **C** Pathway enrichment analysis of G2019S carriers vs non carriers distributed by diagnosis, control on the left and PD on the right. Pathway enrichment was performed with a pre ranked approach from GSEA using Biological Processes. Y axis shows the significant pathways and x axis normalized expression score. Only significant pathways with *q*-value < 0.01 are shown. **D** Pathway enrichment analysis within G2019S carriers, comparing PD-G2019S vs CTRL-G2019S. Pathway enrichment was performed with a pre ranked approach from GSEA using Biological Processes. Y axis shows the significant pathways and x axis normalized expression score. Only significant pathways with *q*-value < 0.01 are shown. **E** Representative western blots of ABCA1 (upper band, ~220 kDa) in primary human monocytes from CTRL and LRRK2-G2019S donors under untreated, IFNγ, or LPS conditions. Ponceau S staining is shown below each blot as the total-protein loading control. Top, IFNγ experiment: lanes are arranged as G2019S+IFNγ, G2019S, CTRL+IFNγ, CTRL (n = 3/group). Middle, LPS experiment: lanes are arranged as G2019S+LPS, G2019S, CTRL+LPS, CTRL (n = 3/group). Bottom, densitometric quantification of ABCA1 signal normalized to the lane-matched Ponceau S signal for each sample. Each point represents one biological replicate; violin plots show the distribution within each group. Fresh monocytes were collected for each stimulation experiment. Statistical annotations are shown on the graphs

We next examined the effect of G2019S mutation, performing DE between G2019S carriers vs. non carriers across conditions (untreated, IFNγ, and LPS). Although the number of significant DEGs was limited due to high variability in human samples and small sample size (Supplementary Tables 19–21), we found an increase in the number of DEGs between carriers vs. non carriers upon stimulation (Fig. 5A and B and S6A). These results highlight how proinflammatory conditions enhance the G2019S mutation’s impact, linking genetic and environmental factors in PD pathogenesis. To deepen the link of genetics and environment, we further explored the interaction between the G2019S mutation carrier status and the two proinflammatory treatments, and noticed statistically significant changes in several genes related to lipid metabolism (Supplementary Tables 22 & 23). An example is *APOL4*, a member of the apolipoprotein L family which plays a role in lipid exchange. We demonstrated that *APOL4* expression is significantly upregulated in G2019S carriers only under LPS but not under the other conditions (Fig. S6B). Another example is the *LDLRAD4* gene, a member of the low-density lipoprotein receptors which has been involved in TGF-beta signaling. This gene is significantly downregulated in G2019S carriers under IFNγ stimulation, but not in baseline or LPS (Fig. S6B). These examples demonstrate that the G2019S mutation exerts context-specific effects on gene expression in human monocytes.

### Human monocytes carrying the G2019S mutation display transcriptomic alterations in lipid metabolism and phagocytosis related genes

To investigate the role of the G2019S mutation in the context of health and disease, we compared transcriptional signatures among carriers with PD versus healthy individuals. Since G2019S is a major risk factor for PD, understanding these signatures may provide insight into pathogenesis. We performed DE analyses comparing: (i) CTRL-G2019S vs. CTRL-WT and (ii) PD-G2019S vs. PD-WT. Proinflammatory stimuli induced more DEGs within these groups than in the untreated condition (Supplementary Tables 24–29). Pathway analysis using GO: BP revealed that G2019S mutation drives dysregulation in signaling pathways related to phagocytosis, vesicle trafficking, lipid metabolism, and immune response when comparing non-manifesting carriers to controls (CTRL-G2019S vs. CTRL-WT) (Fig. 5C, Supplementary Table 30). However, this signal is less apparent in symptomatic individuals (PD-G2019S vs. PD-WT), likely because some pathways overlap with those of sporadic PD, leading to signal saturation. This transcriptional dysregulation is orchestrated by a number of TFs highlighted in Fig. S6C, including *STAT1* and *STAT2* (implicated in the immune response) or *SPI1* and *FOXP1* (implicated in the myeloid differentiation), whose downstream genes are enriched upon G2019S mutation carriers compared to controls (Supplementary Table 31). These results emphasize the impact of G2019S on monocyte transcriptomes, particularly under proinflammatory conditions, underscoring its role in innate immunity and myeloid cell function.

We also investigated transcriptomic changes driving disease among G2019S carriers by comparing PD-G2019S vs. CTRL-G2019S (Supplementary Tables 32–34). Pathway analysis revealed that monocytes from symptomatic patients present down-regulation in pathways associated with immune response and defense. They also show a down-regulation in genes related to lipid metabolism and endocytosis and vesicle trafficking (Fig. 5D, Supplementary Table 35). Nevertheless, when monocytes are stimulated with either IFNγ or LPS, there is shift in directionality, with pathways related to immune response, lipid metabolism, or vesicle transport being upregulated in PD-G2019S vs. CTRL-G2019S. This shift suggests that monocytes from symptomatic patients exhibit a distinct response to proinflammatory stimuli, with LRRK2 potentially mediating these responses.

We were also interested in determining whether this disruption in lipid metabolism pathways, which is observed in both iMGLs and human-derived monocytes, is also observed at the protein level. ABCA1 is a membrane transporter that promotes cholesterol efflux to apoA-I, enabling HDL formation and maintaining cellular lipid homeostasis. In immune cells, such as macrophages and microglia, ABCA1 limits lipid accumulation and dampens pro-inflammatory signaling, thereby supporting resolution of inflammation. Quantification shows that ABCA1 expression is differentially regulated depending on both genotype and stimulus. Under IFNγ stimulation, a significant interaction between genotype and treatment was observed (two-way ANOVA, *p* = 0.0235), indicating that the effect of IFNγ on ABCA1 expression differs between WT and mutant cells. Indeed, in untreated conditions there is a significantly decreased expression of ABCA1 in G2019S-monocytes compared to WT. Moreover, although IFNγ reduces the expression of this transporter in WT cells, this effect is not observed in G2019S monocytes. Thus, this result demonstrates G2019S carriers are not able to modulate expression of ABCA1 under this proinflammatory stimulus. In contrast, LPS treatment did not show a significant interaction (*p* = 0.3245), suggesting a more comparable response between genotypes under this stimulus. Because the IFNγ and LPS validation experiments were performed using independent donor subsets, the untreated WT and G2019S groups in the two graphs serve as within-experiment controls and are not intended for direct comparison across panels; thus, these data support altered stimulus-dependent regulation of ABCA1 in G2019S monocytes, whereas inference on basal ABCA1 differences is limited. Overall, these data indicate that the G2019S mutation selectively alters ABCA1 regulation in response to specific inflammatory conditions. Although further studies are required, our findings indicate that LRRK2-G2019S influences monocyte function and the immune response differently under healthy and disease-related conditions.

## Discussion

The LRRK2-G2019S mutation, a common genetic variant linked to Parkinson’s disease, has been associated with key cellular processes, including phagocytosis, autophagy, inflammation, vesicular trafficking, mitophagy, and lysosomal function [15]. Despite its high expression in immune cells, the role of *LRRK2* in myeloid cells, such as microglia and monocytes, has been less explored. To address this, we examined the transcriptomic impact of LRRK2-G2019S under proinflammatory conditions using iPSC-derived microglia (iMGLs) and patient-derived monocytes.

Our results showed that IFNγ elicited a stronger response in microglia than LPS, an unexpected finding given LPS’s well-established role in microglial activation. Despite this, the responses to both stimuli overlapped significantly, highlighting shared transcriptional programs. LRRK2-G2019S significantly altered the microglial transcriptome, enriching disease-associated microglial signatures (DAM, LAM, ARM). These results show that LRRK2-G2019S iMGLs are capturing the transcriptomic network of different disease associated microglial phenotypes related to neurodegenerative diseases [41].

We subsequently found that G2019S upregulates lipid-related pathways, consistent with genetic studies linking lipid metabolism genes (e.g., *GBA*, *SREBF1*, *ASAH1*) to PD risk. LRRK2-G2019S carriers show altered lipid metabolites in plasma and CSF [42] and postmortem brain tissues show alpha-synuclein colocalized with lipid-rich structures [43], highlighting a key role for lipid homeostasis in microglial function and neurodegeneration [44, 45]. Additionally, LRRK2-G2019S iMGLs upregulate phago-lysosomal genes, overlapping with protein QTLs (pQTLs) in CSF linked to LRRK2 variants [39]. Several studies suggest that LRRK2-G2019S enhances myeloid cell phagocytosis, including uptake of beads and *E. coli* particles [46, 47], while *LRRK2* inhibition reduces microglial engulfment [48]. Nevertheless, our data reveal alterations in phagolysosomal genes, along with changes in lipid transport, storage, and metabolism pathways in LRRK2-G2019S iMGLs, leading to increased accumulation of lipid-rich myelin debris. Since this was not accompanied by increased bead uptake, as also shown in previous studies [49, 50], it suggests a defect in the clearance of lipid debris rather than enhanced uptake.

Furthermore, we observed downregulation of genes associated with mitosis, cell cycle, and proliferation, many of which are regulated by E2F transcription factors. While E2Fs have been implicated in neuronal loss [51–53] and astrocyte proliferation in response to injury [54], their role in myeloid cells remains poorly understood. A recent paper by Belhocine et al. uncovered two context-specific programs of transcriptional regulation of microglial proliferation in vivo, the latter of which involved E2F transcription factors as a regulatory checkpoint to enable microglia to enter the cell cycle [55]. Overall, growth arrest is a feature of senescent microglia, a phenotype also implicated in neurodegeneration [56]. This signature may be also captured by LRRK2-G2019S which reflects dysfunctional microglia that accumulate damage over time.

To complement the use of a single iPSC line in our study, we also included transcriptomic data from patient derived monocytes. While the cohort size is limited, constraining the scope of our conclusions, we observed a notable increase in DEGs in LRRK2-G2019S carriers following inflammatory challenge, reinforcing the interplay between the mutation and environmental factors. Moreover, we observed that patients carrying the LRRK2-G2019S mutation showed a down-regulation in genes related to lipid metabolism, endocytosis and vesicle trafficking, capturing the importance of lipid homeostasis in the disease, as previously shown in our iMGL data. Furthermore, monocytes from LRRK2-G2019S carriers showed reduced expression in pathways linked to immune response and defense, potentially indicating impaired immune function, as reported for other LRRK2 variants [57].

Despite extensive study, the final causal mechanisms underlying neurodegeneration in LRRK2 G2019S carriers remain unresolved. Although this mutation alters key pathways in myeloid cells, including microglia and peripheral monocytes—such as lipid metabolism, inflammatory signaling, and cell proliferation—it is still unclear whether these changes initiate neurodegeneration or arise as secondary responses to ongoing neuronal damage. Dysregulation of lipid homeostasis, particularly sphingolipids, has been shown to disrupt membrane integrity, mitochondrial function, and intracellular signaling in glial cells, thereby impairing autophagy–lysosomal clearance and promoting protein aggregation and progressive neuronal loss [58]. Conversely, accumulating evidence suggests that pathological α-synuclein itself may act upstream by recruiting LRRK2-expressing pro-inflammatory monocytes into the brain, amplifying neuroinflammation and immune-mediated neurodegeneration [59]. In the future, it would be interesting to explore additional components of this phagolysosomal pathway, which sits at the intersection of other important functions including autophagy, macropinocytosis, and efferocytosis. Together, these findings highlight a complex bidirectional relationship between lipid dysregulation, immune activation, and α-synuclein pathology in LRRK2-associated Parkinson’s disease.

Although this study is limited by the use of iPSC-derived cells from a single donor, we mitigated this by including heterozygous and homozygous LRRK2-G2019S carriers and multiple differentiation batches, and integrating patient-derived data. While our iMGL findings were not perfectly recapitulated in human monocytes, such differences may be attributable to the inherent differences between these myeloid cell types, extended in vitro cell culture for our iMGLs, and the effect of age, which is preserved in monocytes. Our findings provide a foundation for future exploration of LRRK2 dysregulation, with expanded sample size, lipidomic profiling and LRRK2 inhibition studies being the next critical steps. Additionally, while our study included monocytes from both sexes, we were unable to assess sex-specific effects due to sample size limitations, an important consideration for future research.

In summary, this study reveals that LRRK2-G2019S induces transcriptomic dysregulation in human microglia and monocytes, particularly in pathways related to lipid metabolism, phago-lysosomal processes, and cell division. These findings highlight the interplay between genetic and environmental factors in PD and reinforce the pivotal role of myeloid cells in disease pathogenesis. Future studies will be essential to unravel the mechanisms by which LRRK2 influences pathways and contributes to the onset and progression of PD.

## Methods

### iPSC culture

Isogenic iPSCs for G2019S (wild-type, heterozygous, and homozygous clones from one donor) were provided by the Cookson Lab (NIH) [30] and all details regarding the origin and CRISPR gene editing are published [30]. These iPSCs (NIAi001-C and NIAi001-D) are derived from the A18945 line (Thermo Scientific, cat # A18945), which itself was derived from CD34 + cord blood. Genotype was validated by Sanger sequencing, and all clones displayed a normal karyotype and standard pluripotent expression markers. iPSCs were maintained in StemFlex medium and cultured on Matrigel-coated 6-well plates. Media was changed every other day, and cells were split using ReLeSR about once per week.

### Generation of iPSC-derived iMGLs

We followed the protocol as described in Abud et al. 2017 [31]. iPSCs were first differentiated to hematopoietic stem cells over a period of 12 days. Briefly, iPSCs were dissociated into a single-cell suspension using TrypLE Select and plated at ~ 500k cells/well onto tissue culture-treated 6-well plates in StemFlex + rock inhibitor. After 24 h (HPC diff: day 1), the media and floating cells were collected, spun, and resuspended in differentiation medium (DM) 1, which consisted of HPC base medium supplemented with FGF2, BMP4, Activin A, LiCl, and rock inhibitor, and replated and cultured under hypoxic (5% O2) conditions. On day 3, cells were switched to DM 2, which included FGF2 and VEGF. On day 5, cells were switched to DM 3, which included FGF2, VEGF, IL6, TPO, SCF, and IL3, and switched back to a normoxic incubator. Cells were supplemented with additional DM3 every other day until floating HPCs were collected between days 11–15 and either frozen in Bambanker Freezing Medium or cultured directly in iMGL medium as described below.

HPCs were plated at 166k/well in a 6-well plate in iMGL medium, consisting of DMEM/F12, GlutaMAX, Non-essential amino acids, B27, N2, ITS, insulin, and MTG, and supplemented with fresh IL-34, M-CSF, and TGFb on each feeding day. Cells were fed every other day with 1mL medium, and were split 1:2 once per week over the course of the 25 day HPC → iMGL differentiation protocol. At day 25–28, cells were supplemented with the above factors along with two maturation factors: CX3CL1 and CD200. Cells were cultured in this medium for 3 days and used for subsequent assays on day 28–31. For RNA-seq and qPCR analysis: matured iMGLs were plated at 500k cells per well of a 6-well plate and treated with 10ng/mL of LPS or 20ng/mL of IFNγ. After 24 h of treatment, cells were washed once with PBS and then lysed directly in the well using RLT buffer with beta-mercaptoethanol (Qiagen RNA-easy Mini Kit) and lysed cells were kept at -80 C and processed together. Each staggered HPC → iMGL differentiation is considered one biological replicate.

### Patient recruitment

This study is part of the MyND (Myeloid cells in Neurodegenerative Diseases) initiative which aims to characterize the immune system (monocytes and microglia) from PD, Alzheimer’s disease and age-matched controls. For this study, we have selected a subset of samples including Controls and PD, with and without the G201S variant in the *LRRK2* gene. Sample collection was performed as part of ongoing research studies at two centers: Bonnie and Tom Strauss Movement Disorder Center at Mount Sinai Beth Israel (MSBI) and Fresco Institute for Parkinson’s and Movement Disorders at New York University (NYUMD). All participants gave informed consent at the respective institutions to contribute samples to this research, and participants included diagnosed PD cases, family members who harbored *LRRK2* G2019S variants, but did not have PD, and non-variant controls.

#### Mount Sinai/Mount Sinai Beth IsraelMovement Disorder (BPMD)

Diagnosis of PD was determined by movement disorder neurologist, when participants met the UK Parkinson’s disease society Brain Bank (UKBB) criteria for probable PD [60], except that family history might be present.Family members participated in genetics research studies who both harbored and did not have the LRRK2 G2019S variant, and did not have known neurodegenerative diseases or auto-immune diseases were collected as controls. Participants were all co-enrolled in Genetics of Parkinson Disease Study and most in U01NS107016-01A1.

#### New York Movement Disorder (NYUMD)

Inclusion criteria followed the United Kingdom Parkinson’s Disease Society Brain Bank Clinical Diagnostic Criteria [61]. A population of age and sex-matched non-affected subjects was enrolled as controls, which did not have known PD at the time of evaluation and no history of any other relevant neurodegenerative disorder.

### PBMC isolation

All experiments were performed with monocytes derived from cryopreserved PBMCs to avoid any technical variability. Blood processing was performed as described before [6]. Briefly, blood was collected in Vacutainer blood collection tubes with acid citrate dextrose (ACD) (BD Biosciences) and was shipped to the Raj laboratory to be processed within the next 2–3 h. Blood was centrifuged at 1,500 g for 15 min, and aliquots of whole blood and plasma were stored at -80 °C for subsequent analysis. Secondly, SepMate tubes were filled with 15 ml Ficoll-Plaque PLUS (GE Healthcare) and 2-fold PBS (Gibco) diluted blood was added and centrifuged 15 min at 1,200 g. After centrifugation, cells were washed with PBS and cryopreserved in 90% FBS (Germini) + 10% DMSO (Sigma Aldrich) at a concentration of 10 million cells/ml in Nalgene cryogenic vials (ThermoScientific). Vials were placed in NalGene.

CryoFreezing containers at -80 °C during 24–72 h, and subsequently placed at liquid nitrogen for storage long-term.

### Monocyte isolation, culture and stimulation

Experiments were performed in 2 independent experimental batches. Monocytes were isolated from cryopreserved PBMCs as follows. Cryopreserved PBMCs were thawed in a water-bath and diluted in 10 ml of media (RPMI + 10% FBS + P/S + Gln) and centrifuged. After washing, PBMCs were sorted to monocytes using CD14^+^ magnetic beads (Miltenyi) in the Manual sorter following the manufacturer’s instructions. Monocyte number and viability was assessed using Countess II Automated Cell counter (Thermo Fisher). After sorting, monocytes were resuspended in supplemented RPMI media at a concentration of 1 million cells/ml. 500,000 cells were plated with the following stimuli: LPS-EK ultrapure (InvivoGen) 10 ng/ml, IFNγ (R&D Systems) 20 ng/ml or baseline (media + PBS). After 24 h of culture at 37 °C and 5% CO2, cells were washed with PBS and pellets were resuspended in 350 µl of RLT + 1% 2-Mercaptoethanol (Sigma Aldrich) and stored at -80 °C.

### RNA isolation and RNAseq

RNA was isolated using RNeasy Mini kit (Qiagen) following the manufacturer’s instructions and including the optional DNase treatment. RNA was stored at -80 °C prior to library preparation. Library preparation and sequencing was performed at Genewiz Inc. using the SMART-Seq v4 Ultra Low input library preparation protocol, which uses poly-A selection. Samples were sequenced with a depth of 30 million 150-bp paired-end reads using Illumina HiSeq 4000 platform.

### RNAseq analysis

#### RNA-seq data processing, quality controls and normalization

RNAseq data was processed as described before [6] In brief, RAPID-nf was used to process FASTQ files. After adapter trimming with trimmomatic (v0.36) [62], STAR (2.7.2a) [63] was used to align samples to the hg38 build (GRCh38.primary_assembly) of the human reference genome with indexes created from GENCODE (v30). For gene expression quantification, we used RSEM (1.3.1) [64]. Sequencing quality and technical metrics were assessed with FASTQC before alignment, and Picard (2.20) and Samtools (v1.9) [65] post-alignment. No samples were removed based on these metrics. Multidimensional reduction using principal component analysis (PCA) and multidimensional scaling (MDS) before and after regressing covariates were used for the identification of outliers. No outliers were removed based on multidimensional reduction. Samples mismatches were identified by the expression of the sex genes *UTY* and *XIST* compared to the reported sex. One sample was removed because of sex mismatch.

Individual gene counts and transcripts per million (TPM) used for downstream analyses were generated using RSEM and assembled to a matrix via the tximport R package. Then, counts per million (CPM) were calculated using cpm() function from the edgeR packing in R. Lowly expressed genes were filtered out, which were defined as having less than one count per million in at least 10% of the samples.

#### Covariate selection using variancePartition

In order to avoid confounders, we used the R package variancePartition [66] to understand the major sources of variation in gene expression. Gene counts were normalized using TMM (trimmed means of M-values) calculated from edgeR, followed by voom transformation, which was used as input for variancePartition. Based on the variancePartition results we selected the covariates which explain the most variance in gene expression to be regressed out in the downstream analysis. For the iMGLs the covariates selected were: *% reads aligned*,* % of duplication + batch of RNA isolation*.

For the human monocytes dataset we controlled variation as follows: *% coding bases + median 3-prime bias + gender + RNA concentration + age + % intergenic bases + the first 4 MDS from the genotype data.*

#### Differential gene expression analysis

For doing differential expression we used the R package limma [67]. The inputs included the count matrix and the covariate file. The data was normalized using TMM values calculated from edgeR, followed by voom transformation. *P*-values were adjusted for multiple comparisons using the Benjamini-Hochberg false discovery rate (FDR) correction.

### Pathway enrichment analysis

Pathway analysis was performed using GSEA [68] focusing on biological processes from GO. The input gene sets were generated ranking genes obtained by differential expression in terms of t-value. We used either the GSEA software or the fgsea function in R. We focused on biological processes from GO. To test specific pathways we used several curated gene lists from the bibliography (Supplementary Table 11). We tested the statistical enrichment using Fisher’s exact test.

In order to explore if genes were enriched for specific transcription factors, we used DoRothEA [69, 70]. We filtered regulons filtered at confidence levels “A” and “B” and performed enrichment using fGSEA with preranked data in terms of t value. Only regulons with adjusted *p*-value < 0.05 were considered.

### DNA isolation and genotyping

DNA was isolated using 1 ml from whole blood using the QiAamp DNA blood Midi kit (Qiagen) following the manufacturer’s instructions. DNA concentration and quality was assessed using nanodrop. For genotyping we used the Illumina Infinium Global Screening Array (GSA). Genetic and Ashkenazi Jewish ancestry was assessed as described before [7]. Additionally, targeted genotyping for specific regions was performed. G2019S variant at the *LRRK2* was outsourced and genotyped in Dr. William Nichol’s laboratory at the Cincinnati Children’s Hospital. Additionally, we also genotyped for the 11 most common GBA variants and for the 3 major APOE isoforms [6].

### Cytokine/chemokine proteome assay

iMGLs were treated with IFNγ or LPS for 24 h and cell supernatant was collected and frozen at -80 °C. Undiluted samples were sent to Eve Technologies and 48 cytokines were measured using the Human Cytokine/Chemokine Panel 48-Plex Discovery Assay Array (HD48A).

### Human myelin isolation

Myelin debris were isolated from frozen human post-mortem brains using the protocol previouslydescribed [71]. A pool of brain pieces from 4 control donors were mixed together to avoid any effect dependent on the individual. Brain tissue was homogenized in 0.32 M sucrose with protease inhibitors (Cell Signaling Technology) and sonicated for 10 min. A sucrose gradient was prepared using 15 ml of 0.85 M sucrose and 15 ml of 0.32 M sucrose containing tissue homogenate and was centrifuged for 40 min at 24,4000 rpm using a Beckman L70 ultracentrifuge, with low acceleration and deceleration. Afterwards, the layer containing myelin was collected, washed with MilliQ water and centrifuged at 24,4000 rpm for 15 min. The pellet was submitted to osmotic shock by resuspending in water and centrifuging at 9800 rpm for 15 min twice. The pellet was then resuspended in 0.32 M sucrose and combined with 0.85 M sucrose in a 1:1 ratio, and centrifuged at 24,4000 rpm for 40 min. The interface was again collected and resuspended in 0.1 M sodium bicarbonate (Sigma-Aldrich). The volume was adjusted to obtain a final protein concentration of 1 µg/ml. Absence of contamination by endotoxins were assessed using a commercial kit following manufacturer instructions (GenScript) and myelin fragments were conjugated with 1:100 pHrodo red dye (Invitrogen) according to the manufacturer’s protocol and stored at − 80 °C.

### Phagocytosis assays

Phagocytic capacity of iMGLs was measured using either red fluorescent carboxylate-modified polystyrene latex beads (Millipore Sigma, 0.9 μm) or pHrodo labeled human myelin. iMGLs were plated at ~ 30,000 cells/well in a matrigel-coated 96-well plate and allowed to attach for 24 h. Cells were then treated with fresh media alone, or media containing 20ng/mL of IFNγ or 10ng/mL of LPS for 24 h. Fresh media was changed the next morning and cells were then treated with carboxylated beads or pH-rodo labeled human myelin for 24 h. Cytochalasin D 2 μm was added as negative control for the final 3 h.

For myelin phagocytosis, phagocytosis was measured by imaging using IncuCyte by monitoring the plates during the 24 h before and after myelin addition. Pictures were taken every hour. Phagocytosis was assessed quantifying red object intensity and normalizing against phase confluence using the IncuCyte ZOOM software as described in detail [71].

For assessing carboxylated-beads uptake, cells were washed once with 1x DPBS at the conclusion of treatment, and incubated in Live/Dead Violet stain (Sigma-Aldrich) for 15 min at 37 C. Cells were then washed again using 1x DPBS and resuspended in 140uL DPBS for flow cytometry using the Attune. After selecting viable cells, the phagocytic index was calculated as the percentage of red fluorescent-positive cells multiplied by the geometric mean of red fluorescence intensity divided by 10 [7]. All treatments were performed with at least 3 technical replicates (each technical replicate as one well of a 96-well plate) and 3 biological replicates (each biological replicate as one differentiation).

### Proliferation capacity

In order to assess the proliferation capacity, cells were monitored for 24 h using IncuCyte. Proliferation of cells was quantified as phase confluence using IncuCyte ZOOM software. Experiments were performed with at least 3 technical replicates (each technical replicate as one well of a 96-well plate) and 3 biological replicates (each biological replicate as one differentiation).

### Western blotting

Primary human monocyte lysates from control and LRRK2-PD (G2019S) donors were analyzed under untreated and stimulated conditions. IFNγ and LPS experiments were performed on separate membranes run in parallel. For each membrane, four groups were included (control untreated, control stimulated, LRRK2 untreated, LRRK2 stimulated; *n* = 3 donors/group). Cell lysates were generated using Roche cOmplete Lysis-M buffer (Millipore-Sigma) with protease inhibitor cocktails (Millipore-Sigma). Protein concentration was estimated using the Qubit BR protein assay (Invitrogen) in a Qubit 4 Fluorometer. For Western blot analysis, 0.8 µg total protein was denatured under reducing conditions in NuPage LDS sample buffer (ThermoFisher) by heating for 5 min at 80 °C before loading onto a 18-well 4–12% Criterion XT Bis-Tris precast gels (BioRad #3450124), then transferred to a PVDF membrane (0.2 μm; Trans-blot turbo, Bio-Rad) using the iBlot 2 dry blotting system (Invitrogen). Membranes were blocked for 1 h at RT in 5% BSA in TBS containing 0.1% Tween-20 (Fisher Scientific; TBS-T). Membranes were then incubated with anti-ABCA1 (1:1000, NB400-105, Novus/Bio-Techne) in 5% BSA/TBS-T overnight at 4 °C. Following overnight incubation, membranes were washed six times, 5 min in TBST by incubation with HRP-conjugated anti-rabbit IgG secondary antibody (Cell Signaling Technology, #7074) for 45 min at room temperature. Signal was detected using SuperSignal West Femto Maximum Sensitivity Substrate (ThermoFisher). Band intensities were quantified in Fiji/ImageJ. For each target, band signal was measured after local background correction. To account for differences in total protein load and transfer efficiency, the signal from each lane was normalized to the Ponceau S signal of the corresponding lane/sample, using lane-wise total protein as the normalization factor (Sander et al.).

### Statistical analysis

All statistical analyses and figure generation were performed using R (version 4.5.2). Enrichment analyses were conducted using Fisher’s exact test. Differential gene expression analysis was performed using the limma package, and resulting *p*-values were adjusted for multiple testing using the false discovery rate (FDR) method. Pathway and regulon enrichment analyses were carried out using *fgsea*, and statistical significance was assessed based on the adjusted *p*-values reported by the method. Unless otherwise stated, adjusted *p*-values < 0.05 were considered statistically significant. In functional assays, all analysis was performed using R (version 4.0.5) in RStudio (version 1.3.1073). Student’s t-tests (paired) were used for pairwise comparisons. ANOVAs were used for multiple comparisons, followed by Tukey or Dunnett’s post hoc tests when appropriate. Linear mixed models were used when comparing genotypes and treatments over time for live-imaging analyses. A Type 1 error significance level of 0.05 was used to determine statistical significance for all calculations.

## Supplementary Information

Supplementary Material 1.

Supplementary Material 2.

Supplementary Material 3: Figures 1-6

## Data Availability

The raw RNA-seq data from induced microglia-like cells (iMGLs) can be accessed on GEO (GSE240907), while stimulated monocyte data is available on dbGAP (study accession ID: phs002400.v1.p1) through this link: https://www.ncbi.nlm.nih.gov/projects/gap/cgi-bin/study.cgi?study_id=phs002400.v1.p1.

## References

[1] Polymeropoulos MH, et al. Mapping of a gene for Parkinson’s disease to chromosome 4q21-q23. Science. 1996;274:1197–9. 10.1126/science.274.5290.1197 8895469

[2] Nichols WC, et al. Genetic screening for a single common LRRK2 mutation in familial Parkinson’s disease. Lancet. 2005;365:410–2. 10.1016/S0140-6736(05)17828-3 15680455

[3] Ibáñez P, et al. Causal relation between α-synuclein locus duplication as a cause of familial Parkinson’s disease. Lancet. 2004;364:1169–71. 10.1016/S0140-6736(04)17104-3 15451225

[4] Chartier-Harlin M-C, et al. α-synuclein locus duplication as a cause of familial Parkinson’s disease. Lancet. 2004;364:1167–9. 10.1016/S0140-6736(04)17103-1 15451224

[5] Nalls MA, et al. Identification of novel risk loci, causal insights, and heritable risk for Parkinson’s disease: a meta-analysis of genome-wide association studies. Lancet Neurol. 2019;18:1091–102. 10.1016/S1474-4422(19)30320-5 PMC8422160 31701892

[6] Navarro E, et al. Dysregulation of mitochondrial and proteolysosomal genes in Parkinson’s disease myeloid cells. Nat Aging. 2021;1:850–63. 10.1038/s43587-021-00110-x PMC8728893 35005630

[7] de Lopes K. Genetic analysis of the human microglial transcriptome across brain regions, aging and disease pathologies. Nat Genet. 2022;54:4–17. 10.1038/s41588-021-00976-y PMC9245609 34992268

[8] Li YI, Wong G, Humphrey J, Raj T. Prioritizing Parkinson’s disease genes using population-scale transcriptomic data. Nat Commun. 2019;10:994. 10.1038/s41467-019-08912-9 PMC6397174 30824768

[9] Gagliano SA et al. Genomics implicates adaptive and innate immunity in Alzheimer’s and Parkinson’s diseases. Annals of Clinical and Translational Neurology. 2016;3:924–933. Preprint at 10.1002/acn3.369. PMC5224821 28097204

[10] Reynolds RH, et al. Moving beyond neurons: the role of cell type-specific gene regulation in Parkinson’s disease heritability. npj Parkinson’s Disease. 2019;5. Preprint at 10.1038/s41531-019-0076-6. PMC6470136 31016231

[11] Andersen MS, et al. Heritability Enrichment Implicates Microglia in Parkinson’s Disease Pathogenesis. Ann Neurol. 2021;89:942–51. 10.1002/ana.26032 PMC9017316 33502028

[12] Raj T, et al. Polarization of the effects of autoimmune and neurodegenerative risk alleles in leukocytes. Science. 2014;344:519–23. 10.1126/science.1249547 PMC4910825 24786080

[13] Blauwendraat C, Nalls MA, Singleton AB. The genetic architecture of Parkinson’s disease. Lancet Neurol. 2020;19:170–8. 10.1016/S1474-4422(19)30287-X PMC8972299 31521533

[14] Schilder BM, Raj T. Fine-mapping of Parkinson’s disease susceptibility loci identifies putative causal variants. Hum Mol Genet. 2022;31:888–900. 10.1093/hmg/ddab294 PMC8947317 34617105

[15] Kluss JH, Mamais A, Cookson MR. LRRK2 links genetic and sporadic Parkinson’s disease. Biochem Soc Trans. 2019;47:651–61. 10.1042/BST20180462 PMC6563926 30837320

[16] Berwick DC, Heaton GR, Azeggagh S, Harvey K. LRRK2 Biology from structure to dysfunction: research progresses, but the themes remain the same. Mol Neurodegener. 2019;14:49. 10.1186/s13024-019-0344-2 PMC6925518 31864390

[17] Bardien S, Lesage S, Brice A, Carr J. Genetic characteristics of leucine-rich repeat kinase 2 (LRRK2) associated Parkinson’s disease. Parkinsonism & Related Disorders. 2011;17:501–508. Preprint at 10.1016/j.parkreldis.2010.11.008. 21641266

[18] Thaler A, Ash E, Gan-Or Z, Orr-Urtreger A, Giladi N. The LRRK2 G2019S mutation as the cause of Parkinson’s disease in Ashkenazi Jews. Journal of Neural Transmission. 2009;116:1473–1482. Preprint at 10.1007/s00702-009-0303-0. 19756366

[19] Thévenet J, Gobert P, van Huijsduijnen RH, Wiessner R, C., Sagot YJ. Regulation of LRRK2 expression points to a functional role in human monocyte maturation. PLoS ONE. 2011;6:e21519. 10.1371/journal.pone.0021519 PMC3124520 21738687

[20] Gillardon F, Schmid R, Draheim H. Parkinson’s disease-linked leucine-rich repeat kinase 2(R1441G) mutation increases proinflammatory cytokine release from activated primary microglial cells and resultant neurotoxicity. Neuroscience. 2012;208:41–48. Preprint at 10.1016/j.neuroscience.2012.02.001. 22342962

[21] Moehle MS, et al. LRRK2 inhibition attenuates microglial inflammatory responses. J Neurosci. 2012;32:1602–11. 10.1523/JNEUROSCI.5601-11.2012 PMC3532034 22302802

[22] Lee H, James WS, Cowley SA. LRRK2 in peripheral and central nervous system innate immunity: its link to Parkinson’s disease. Biochem Soc Trans. 2017;45:131–9. 10.1042/BST20160262 PMC5652224 28202666

[23] Agalliu I, et al. Higher frequency of certain cancers in LRRK2 G2019S mutation carriers with Parkinson disease: a pooled analysis. JAMA Neurol. 2015;72:58–65. 10.1001/jamaneurol.2014.1973 PMC4366130 25401981

[24] Ryan KJ et al. A human microglia-like cellular model for assessing the effects of neurodegenerative disease gene variants. Sci Transl Med. 2017;9. 10.1126/scitranslmed.aai7635 PMC5945290 29263232

[25] Gardet A, et al. LRRK2 Is Involved in the IFN-γ Response and Host Response to Pathogens. J Immunol. 2010;185:5577–85. 10.4049/jimmunol.1000548 PMC3156100 20921534

[26] Cook DA, et al. LRRK2 levels in immune cells are increased in Parkinson’s disease. NPJ Parkinsons Dis. 2017;3:11. 10.1038/s41531-017-0010-8 PMC5459798 28649611

[27] Kuss M, Adamopoulou E, Kahle PJ. Interferon-γ induces leucine-rich repeat kinase LRRK2 via extracellular signal-regulated kinase ERK5 in macrophages. Journal of Neurochemistry. 2014;129:980–987. Preprint at 10.1111/jnc.12668. 24479685

[28] Hakimi M et al. Parkinson’s disease-linked LRRK2 is expressed in circulating and tissue immune cells and upregulated following recognition of microbial structures. Journal of Neural Transmission. 2011;118:795–808. Preprint at 10.1007/s00702-011-0653-2. PMC3376651 21552986

[29] Langston RG, et al. Association of a common genetic variant with Parkinson’s disease is mediated by microglia. Sci Transl Med. 2022;14:eabp8869. 10.1126/scitranslmed.abp8869 PMC9809150 35895835

[30] Beylina A, Langston RG, Rosen D, Reed X, Cookson MR. Generation of fourteen isogenic cell lines for Parkinson’s disease-associated leucine-rich repeat kinase (LRRK2). Stem Cell Res. 2021;53:102354. Preprint at 10.1016/j.scr.2021.102354. PMC8314873 34087985

[31] Abud EM et al. iPSC-Derived Human Microglia-like Cells to Study Neurological Diseases. Neuron. 2017;94:278–293.e9. Preprint at 10.1016/j.neuron.2017.03.042. PMC5482419 28426964

[32] Fairfax BP, et al. Innate immune activity conditions the effect of regulatory variants upon monocyte gene expression. Science. 2014;343:1246949. 10.1126/science.1246949 PMC4064786 24604202

[33] Shen L, GeneOverlap. An R package to test and vi- sualize gene overlaps. http://bioconductor.statistik.tu-dortmund.de/packages/3.7/bioc/vignettes/GeneOverlap/inst/doc/GeneOverlap.pdf.

[34] Keren-Shaul H et al. A Unique Microglia Type Associated with Restricting Development of Alzheimer’s Disease. Cell. 2017;169:1276–1290.e17. Preprint at 10.1016/j.cell.2017.05.018. 28602351

[35] Hasselmann J, et al. Development of a Chimeric Model to Study and Manipulate Human Microglia In Vivo. Neuron. 2019;103:1016–e103310. 10.1016/j.neuron.2019.07.002 PMC7138101 31375314

[36] McQuade A, et al. Gene expression and functional deficits underlie TREM2-knockout microglia responses in human models of Alzheimer’s disease. Nat Commun. 2020;11:5370. 10.1038/s41467-020-19227-5 PMC7584603 33097708

[37] Sala Frigerio C, et al. The Major Risk Factors for Alzheimer’s Disease: Age, Sex, and Genes Modulate the Microglia Response to Aβ Plaques. Cell Rep. 2019;27:1293–e13066. 10.1016/j.celrep.2019.03.099 PMC7340153 31018141

[38] Jaitin DA, et al. Lipid-Associated Macrophages Control Metabolic Homeostasis in a Trem2-Dependent Manner. Cell. 2019;178:686–e69814. 10.1016/j.cell.2019.05.054 PMC7068689 31257031

[39] Phillips B, et al. Proteome wide association studies of LRRK2 variants identify novel causal and druggable proteins for Parkinson’s disease. NPJ Parkinsons Dis. 2023;9:107. 10.1038/s41531-023-00555-4 PMC10329646 37422510

[40] Ahmadi Rastegar D, et al. Effect of LRRK2 protein and activity on stimulated cytokines in human monocytes and macrophages. NPJ Parkinsons Dis. 2022;8:34. 10.1038/s41531-022-00297-9 PMC8960803 35347144

[41] Paolicelli RC, Angiari S. Microglia immunometabolism: From metabolic disorders to single cell metabolism. Semin Cell Dev Biol. 2019;94:129–37. 10.1016/j.semcdb.2019.03.012 30954657

[42] Galper J, et al. Lipid pathway dysfunction is prevalent in patients with Parkinson’s disease. Brain. 2022;145:3472–87. 10.1093/brain/awac176 PMC9586542 35551349

[43] Shahmoradian SH, et al. Lewy pathology in Parkinson’s disease consists of crowded organelles and lipid membranes. Nat Neurosci. 2019;22:1099–109. 10.1038/s41593-019-0423-2 31235907

[44] Marschallinger J, et al. Lipid-droplet-accumulating microglia represent a dysfunctional and proinflammatory state in the aging brain. Nat Neurosci. 2020;23:194–208. 10.1038/s41593-019-0566-1 PMC7595134 31959936

[45] Bartels T, De Schepper S, Hong S. Microglia modulate neurodegeneration in Alzheimer’s and Parkinson’s diseases. Science. 2020;370:66–9. 10.1126/science.abb8587 33004513

[46] Kim KS, et al. Regulation of myeloid cell phagocytosis by LRRK2 via WAVE2 complex stabilization is altered in Parkinson’s disease. Proc Natl Acad Sci U S A. 2018;115:E5164–73. 10.1073/pnas.1718946115 PMC5984500 29760073

[47] Panagiotakopoulou V, et al. Interferon-γ signaling synergizes with LRRK2 in neurons and microglia derived from human induced pluripotent stem cells. Nat Commun. 2020;11:5163. 10.1038/s41467-020-18755-4 PMC7560616 33057020

[48] Marker DF, et al. LRRK2 kinase inhibition prevents pathological microglial phagocytosis in response to HIV-1 Tat protein. J Neuroinflammation. 2012;9:261. 10.1186/1742-2094-9-261 PMC3538654 23190742

[49] Ohtonen S, et al. Human iPSC-derived microglia carrying the LRRK2-G2019S mutation show a Parkinson’s disease related transcriptional profile and function. Sci Rep. 2023;13:22118. 10.1038/s41598-023-49294-9 PMC10719377 38092815

[50] Moehle MS, et al. The G2019S LRRK2 mutation increases myeloid cell chemotactic responses and enhances LRRK2 binding to actin-regulatory proteins. Hum Mol Genet. 2015;24:4250–67. 10.1093/hmg/ddv157 PMC4492391 25926623

[51] Höglinger GU, et al. The pRb/E2F cell-cycle pathway mediates cell death in Parkinson’s disease. Proc Natl Acad Sci U S A. 2007;104:3585–90. 10.1073/pnas.0611671104 PMC1805567 17360686

[52] Alvira D, et al. Activation of the calpain/cdk5/p25 pathway in the girus cinguli in Parkinson’s disease. Parkinsonism Relat Disord. 2008;14:309–13. 10.1016/j.parkreldis.2007.09.005 17977053

[53] Hou ST, et al. The transcription factor E2F1 modulates apoptosis of neurons. J Neurochem. 2000;75:91–100. 10.1046/j.1471-4159.2000.0750091.x 10854251

[54] Swiss VA, Casaccia P. Cell-context specific role of the E2F/Rb pathway in development and disease. Glia. 2010;58:377–90. 10.1002/glia.20933 PMC2882865 19795505

[55] Belhocine S, et al. Context-dependent transcriptional regulation of microglial proliferation. Glia. 2022;70:572–89. 10.1002/glia.24124 34862814

[56] Rim C, You M-J, Nahm M, Kwon M-S. Emerging role of senescent microglia in brain aging-related neurodegenerative diseases. Transl Neurodegener. 2024;13:10. 10.1186/s40035-024-00402-3 PMC10877780 38378788

[57] Wallings RL, et al. The R1441C-Lrrk2 mutation induces myeloid immune cell exhaustion in an age- and sex-dependent manner in mice. Sci Transl Med. 2024;16:eadl1535. 10.1126/scitranslmed.adl1535 PMC12718673 39504353

[58] Islam MN, et al. Sphingolipid signaling and metabolism in neuronal and glial cells: Implications for cerebrovascular and neurodegenerative disorders. Aging Dis. 2025. 10.14336/AD.2025.1024. 41400579

[59] Xu E, et al. Pathological α-synuclein recruits LRRK2 expressing pro-inflammatory monocytes to the brain. Mol Neurodegener. 2022;17:7. 10.1186/s13024-021-00509-5 PMC8751347 35012605

[60] Daniel SE, Lees AJ. Parkinson’s Disease Society Brain Bank, London: overview and research. J Neural Transm Suppl. 1993;39:165–72. 8360656

[61] Hughes AJ, Ben-Shlomo Y, Daniel SE, Lees AJ. UK Parkinson’s disease society brain bank clinical diagnostic criteria. J Neurol Neurosurg Psychiatry. 1992;55:e4.

[62] Bolger AM, Lohse M, Usadel B. Trimmomatic: a flexible trimmer for Illumina sequence data. Bioinformatics. 2014;30:2114–20. 10.1093/bioinformatics/btu170 PMC4103590 24695404

[63] Dobin A, et al. STAR: ultrafast universal RNA-seq aligner. Bioinformatics. 2013;29:15–21. 10.1093/bioinformatics/bts635 PMC3530905 23104886

[64] Li B, Dewey CN. RSEM: accurate transcript quantification from RNA-Seq data with or without a reference genome. BMC Bioinformatics. 2011;12:323. 10.1186/1471-2105-12-323 PMC3163565 21816040

[65] Li H, et al. The Sequence Alignment/Map format and SAMtools. Bioinformatics. 2009;25:2078–9. 10.1093/bioinformatics/btp352 PMC2723002 19505943

[66] Hoffman GE, Schadt EE, variancePartition. Interpreting drivers of variation in complex gene expression studies. Preprint at 10.1101/040170. PMC5123296 27884101

[67] Ritchie ME, et al. limma powers differential expression analyses for RNA-sequencing and microarray studies. Nucleic Acids Res. 2015;43:e47. 10.1093/nar/gkv007 PMC4402510 25605792

[68] Subramanian A, et al. Gene set enrichment analysis: a knowledge-based approach for interpreting genome-wide expression profiles. Proc Natl Acad Sci U S A. 2005;102:15545–50. 10.1073/pnas.0506580102 PMC1239896 16199517

[69] Holland CH, Szalai B, Saez-Rodriguez J. Transfer of regulatory knowledge from human to mouse for functional genomics analysis. Biochim Biophys Acta Gene Regul Mech. 2020;1863:194431. 10.1016/j.bbagrm.2019.194431 31525460

[70] Garcia-Alonso L, Holland CH, Ibrahim MM, Turei D, Saez-Rodriguez J. Benchmark and integration of resources for the estimation of human transcription factor activities. Genome Res. 2019;29:1363–75. 10.1101/gr.240663.118 PMC6673718 31340985

[71] Machlovi SI, et al. APOE4 confers transcriptomic and functional alterations to primary mouse microglia. Neurobiol Dis. 2022;164:105615. 10.1016/j.nbd.2022.105615 PMC8934202 35031484

