# Supplementary material for: LRRK2 G2019S variant is associated with transcriptional changes in Parkinson’s disease human myeloid cells under proinflammatory environment
Source: J Neuroinflammation. 2026 Aug 8;23:364. doi: 10.1186/s12974-026-03967-0 (PMC13733072; doi:10.1186/s12974-026-03967-0)

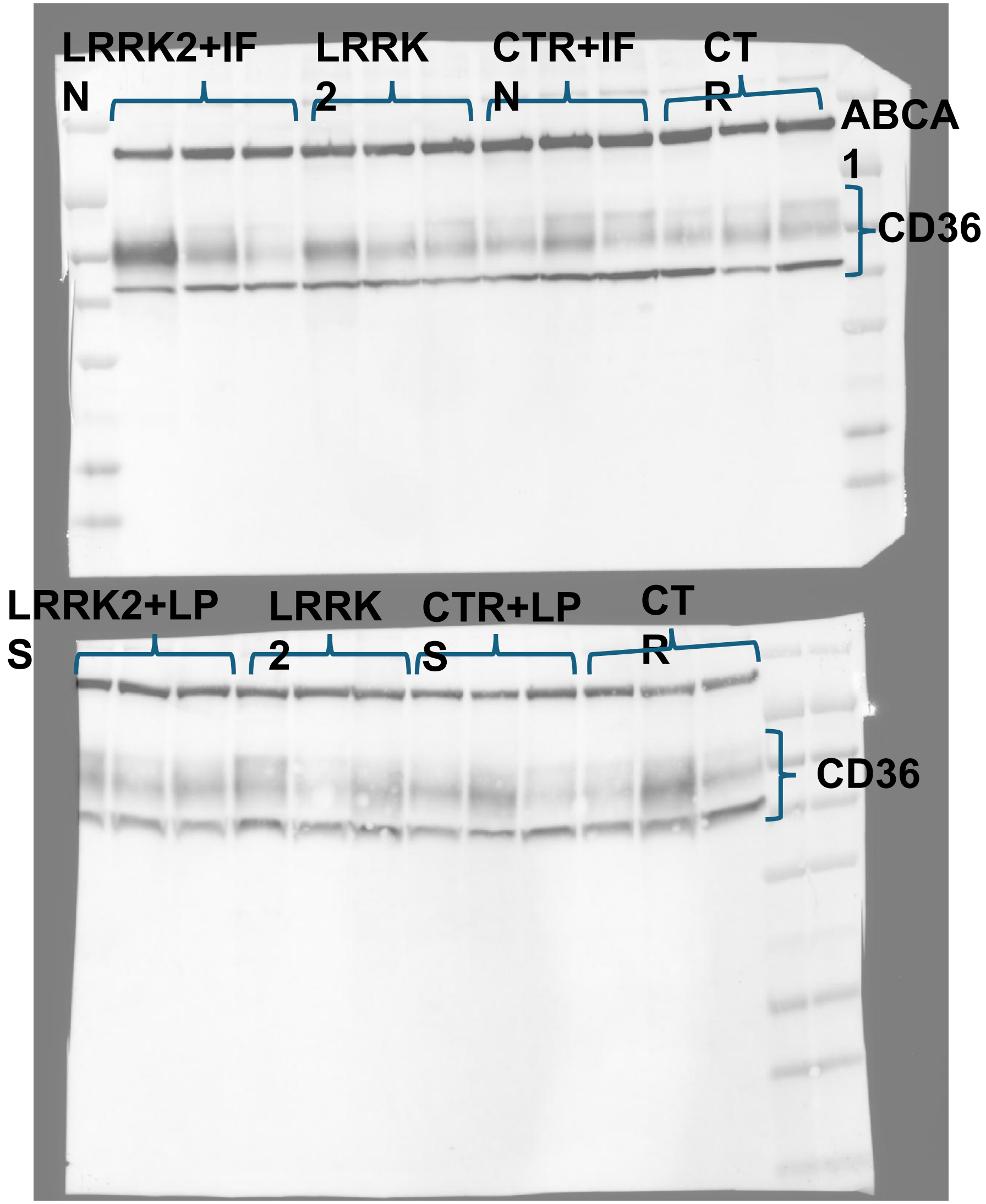
LRRK2+IFN
LRRK2
CTR+IFN
CTR
ABCA1
CD36
LRRK2+LPS
LRRK2
CTR+LPS
CTR
CD36

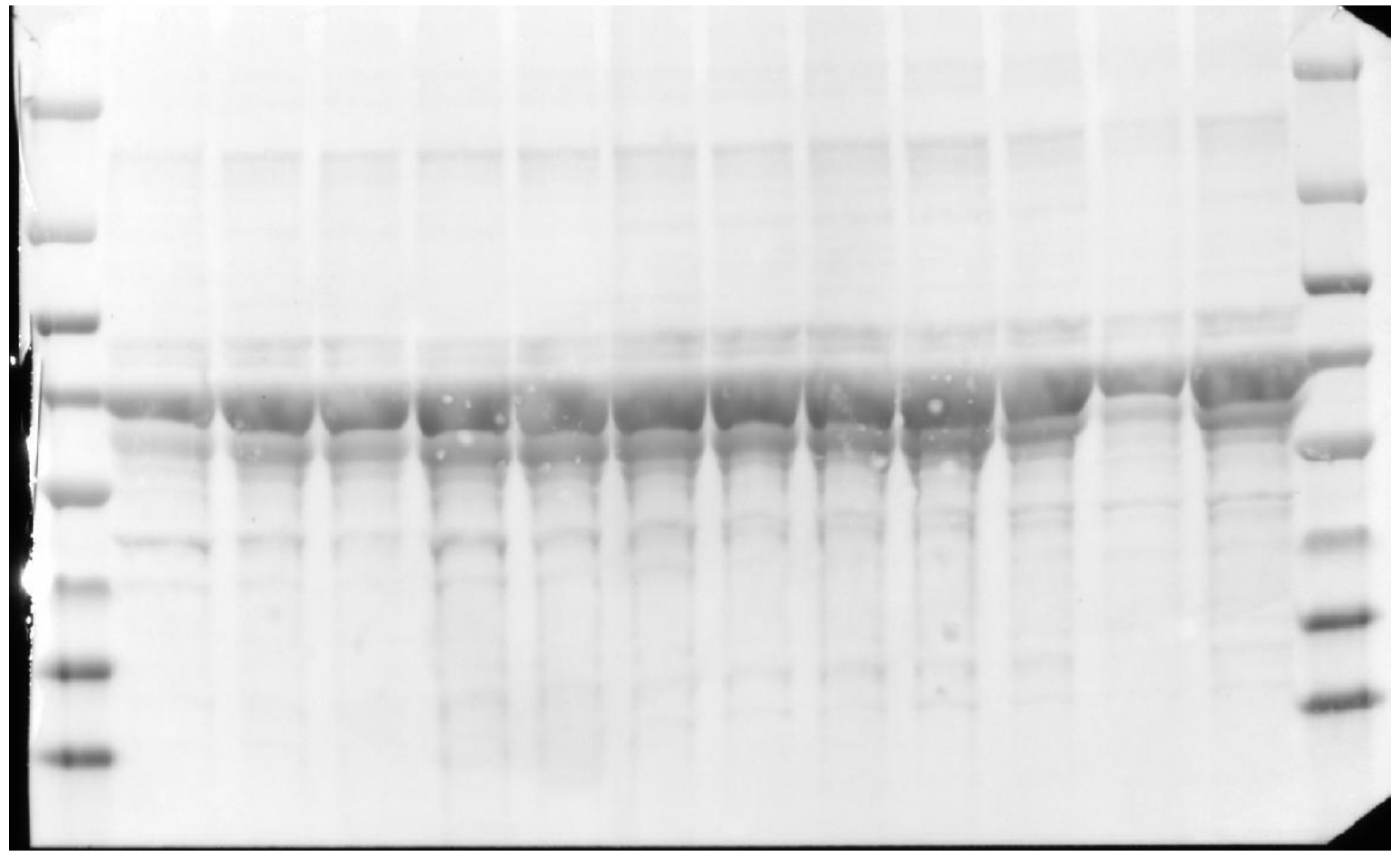

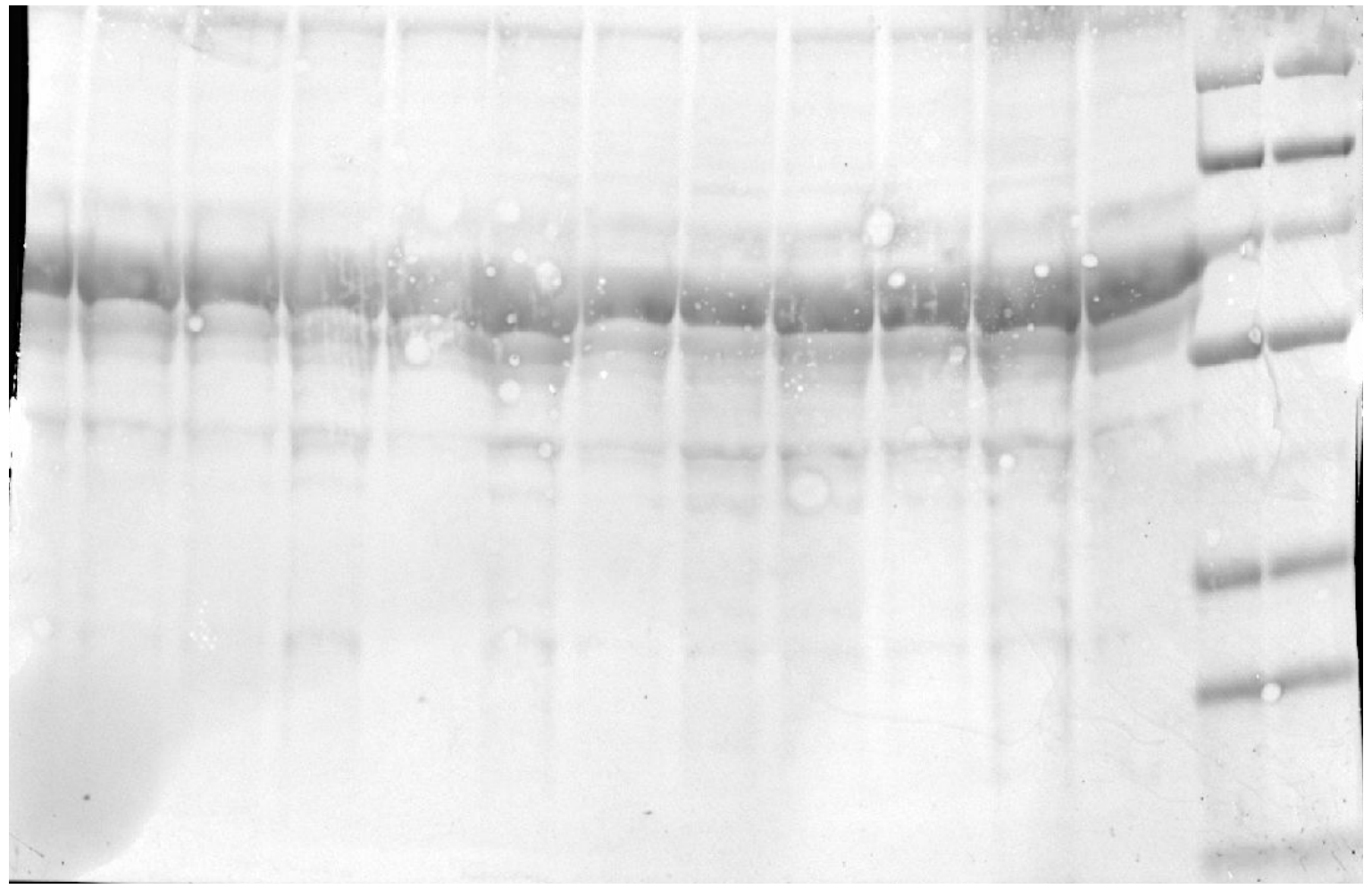

Supplement: Supplementary file 1 [file 12974_2026_3967_MOESM2_ESM.pdf]
