# Supplementary material for: LRRK2 G2019S variant is associated with transcriptional changes in Parkinson’s disease human myeloid cells under proinflammatory environment
Source: J Neuroinflammation. 2026 Aug 8;23:364. doi: 10.1186/s12974-026-03967-0 (PMC13733072; doi:10.1186/s12974-026-03967-0)
Supplement: Supplementary file 2 [file 12974_2026_3967_MOESM3_ESM.docx]

**Supplementary Figures**

**Supplementary Figure 1. Human induced microglial cells (iMGLs) transcriptomic and cytokine analysis.** (A) Principal component analysis (PCA) of the 27 iMGL samples after regressing out technical covariates. Each dot represents a sample and is colored by genotype (left) or treatment (right). (B) qPCR validation of the expression of IFIT1, IL-1b, LRRK2 and TNFa in iMGLs (n=3). (C) Visualization of pathway dysregulation upon IFNγ (left) and LPS (right) treatment using Cytoscape.

**Supplementary Figure 2. Transcriptomic dysregulation in LRRK2-G2019S het and hom vs LRRK2-WT iMGLs.** Volcano plots showing differential expression between G2019S/+ (upper plots) or G2019S/G2019S (bottom plots) vs LRRK2-WT under baseline (left), IFNγ (medium) or LPS (right). Plots show the FC of genes (x axis, log_2_ scale) and their *P* value significance (y axis, –log_10_ scale). DEGs at FDR < 0.05 are highlighted in red (upregulated genes) and blue (downregulated genes). Top DEGs are shown. A moderated *t*-statistic (two-sided) was used as the statistical test.

**Supplementary Figure 3. Human derived monocytes transcriptomic analysis.** (A) Experimental workflow for the generation of monocyte transcriptomic profiles. (B) Tables describing the samples included in the study. (C) Expression of LRRK2 in human derived monocytes upon the different treatments in CTRL-WT (n=9), CTRL-G2019S (n=6), PD-WT (n=12) and PD-G2019S (n=8). Adjusted gene expression levels after normalization are shown. (D) qPCR validation of LRRK2 expression in control monocytes upon the different stimulations (n=6). Boxplots: the line represents the median. The boxes extend from the 25th - 75th percentile.

**Supplementary Figure 4. Quality control in monocyte transcriptomic analysis.** (A) Violin plot showing the % of variance (y axis) explained by known technical and biological covariates (x axis) by variancePartition. (B) Principal component analysis (PCA) of the samples (105 samples from 35 independent donors) before and (C) after regressing out covariates. Each dot represents a sample. Samples are colored by percent of coding bases (left) and treatment (right). (E) PCA showing the expression of the male marker *UTR* and the female marker *XIST*. Samples are colored by sex, showing no sex mismatches. (F) Heatmap of the expression (log_2_(TPM +1)) of marker genes of different blood cell types (represented by colors). Gene markers are represented in the y axis and samples in the x axis (n=105).

**Supplementary Figure 5. Transcriptomic dysregulation in human monocytes upon IFNγ and LPS stimulation**. (A) Volcano plot showing differential expression between IFNγ and (B) LPS stimulation. Plots show the FC of genes (x axis, log_2_ scale) and their *P* value significance (y axis, –log_10_ scale). DEGs at FDR < 0.05 are highlighted in red (upregulated genes) and blue (downregulated genes). A moderated *t*-statistic (two-sided) was used as the statistical test. (C) Heatmap showing the logFC of genes associated with PD or (D) LRRK2 interactors. The heatmap shows a selection of genes (y axis) across samples (x axis), clustered by genotype. Blue represents logFC < 0 and red represents logFC > 0.

**Supplementary Figure 6. Transcriptomic dysregulation in monocytes of G2019S carriers upon diagnosis in IFNγ and LPS stimulation**. (A) Density plot showing the p-value distribution of LRRK2-G2019S carriers vs non-carriers upon the different stimulations (represented by colors). (B) Examples of selected DEGs (*APOL4* on the left and *LDLRAD4* on the right) in which there is significant interaction between treatment and genotype. Adjusted expression of *voom* normalized counts after regression of covariates is shown. In boxplots, the line represents the median and boxes extend from the 25th to the 75th percentile. P-values calculated with Wilcoxon test. (C) Transcription factor enrichment in human derived monocytes of CTRL-G2019S vs CTRL-WT (left) and PD-G2019S vs PD-WT (right) upon the different stimulations (represented by colors). Transcription factors are expressed in the y axis and normalized expression score in the x axis. Only regulons significantly enriched at a q-value < 0.05 are shown. (D) Transcription factor enrichment in human derived monocytes of PD-G2019S vs CTRL-G2019S upon the different stimulations. Transcription factors are expressed in the y axis and normalized expression score in the x axis. Only regulons significantly enriched at a q-value < 0.05 are shown.
